# Vicarious structural racism and infant health disparities in Michigan: The Flint Water Crisis

**DOI:** 10.3389/fpubh.2022.954896

**Published:** 2022-09-06

**Authors:** Kristi L. Allgood, Jasmine A. Mack, Nicole L. Novak, Cleopatra M. Abdou, Nancy L. Fleischer, Belinda L. Needham

**Affiliations:** ^1^Department of Epidemiology, Center for Social Epidemiology and Population Health, University of Michigan School of Public Health, Ann Arbor, MI, United States; ^2^Department of Biostatistics, University of Michigan School of Public Health, Ann Arbor, MI, United States; ^3^Department of Community and Behavioral Health, University of Iowa College of Public Health, Iowa City, IA, United States; ^4^Department of Children, Youth, and Families, Suzanne Dworak-Peck School of Social Work, University of Southern California, Los Angeles, CA, United States

**Keywords:** vicarious racism, structural racism, Flint Water Crisis, birth records, birth outcomes, racial disparities

## Abstract

Building on nascent literature examining the health-related effects of vicarious structural racism, we examined indirect exposure to the Flint Water Crisis (FWC) as a predictor of birth outcomes in Michigan communities outside of Flint, where residents were not directly exposed to lead-contaminated water. Using linear regression models, we analyzed records for all singleton live births in Michigan from 2013 to 2016, excluding Flint, to determine whether birth weight (BW), gestational age (GA), and size-for-gestational-age (SzGA) decreased among babies born to Black people, but not among babies born to White people, following the highly publicized January 2016 emergency declaration in Flint. In adjusted regression models, BW and SzGA were lower for babies born to both Black and White people in the 37 weeks following the emergency declaration compared to the same 37-week periods in the previous 3 years. There were no racial differences in the association of exposure to the emergency declaration with BW or SzGA. Among infants born to Black people, GA was 0.05 weeks lower in the 37-week period following the emergency declaration versus the same 37-week periods in the previous 3 years (95% CI: −0.09, −0.01; *p* = 0.0177), while there was no change in GA for infants born to White people following the emergency declaration (95% CI: −0.01, 0.03; *p* = 0.6962). The FWC, which was widely attributed to structural racism, appears to have had a greater impact, overall, on outcomes for babies born to Black people. However, given the frequency of highly publicized examples of anti-Black racism over the study period, it is difficult to disentangle the effects of the FWC from the effects of other racialized stressors.

## Introduction

Black people have higher risk of pregnancy complications and adverse birth outcomes than other people in the US; however, the mechanisms are not fully understood (1). In 2018, infants delivered by Black people were two times more likely than infants delivered by White people to weigh <5.5 pounds at birth and were 1.5 times more likely to be delivered preterm (2). Differences in birthweight and gestational age contribute to racial disparities in infant mortality and other adverse health outcomes later in life (3, 4). Black/White disparities in birth outcomes are not fully explained by differences in biobehavioral risk factors, such as age, access to prenatal care, and smoking (5, 6), nor are they explained by differences in interpersonal and community-level risk factors, like discrimination, housing insecurity, and neighborhood crime (3, 7, 8). This study builds on the small, but growing body of research examining health-related effects of *vicarious structural racism*, defined as witnessing the effects of racist structural conditions (e.g., racially segregated communities, disparities in incarceration, etc.) or practices (e.g., hazardous waste facilities in minority communities, racial profiling by law enforcement, etc.) on members of one's own racial/ethnic group (9–13). We examine indirect exposure to the Flint Water Crisis (FWC) as a predictor of birth outcomes in Michigan communities outside of Flint and examine differences in associations by race.

Many researchers have posited that racism is a major mechanism by which poor birth outcomes develop (11, 14–24). Most research has focused on interpersonal racism, or circumstances in which an individual is directly treated unfairly because of their race/ethnicity by another individual (25, 26). This is problematic because it ignores the pervasive, but less visible exposure to structural racism, a more common form of racism that does not target an individual, but rather reflects policies that differentially affect one group over another, and may be more detrimental to one's health than interpersonal discrimination (17, 25, 27–35).

Because structural racism is intertwined into the social order of American life, it is a ubiquitous and often ignored exposure, making it very challenging to study (28, 30). One way to examine larger macro discrimination-related stressors is to compare health outcomes before and after major racialized events—a situation in which marginalized groups are more affected than non-marginalized groups. Historically, events that are experienced acutely, or chronically, and become racialized, either through intent (e.g., failing to safely change the drinking water source in Flint, Michigan) or by society's response to an event (e.g., 9/11 attacks or Hurricane Katrina), are prime for such study. These major events that often occur without warning may produce an immediate threat to one's well-being (9, 36, 37). Additionally, the societal response, or lack of a response, may affect longer-term health due to fear, worry, or a reminder of one's place in the racial hierarchy (25, 38–42).

Several studies that incorporate a quasi-experimental design have demonstrated that after major stressful and often racialized events, birth outcomes are substantially worse regardless of whether the event was experienced directly (43–57) or indirectly (vicariously) (9, 58–66). However, some studies have shown mixed results (44, 48, 51, 57) or no association between stressful events and birth outcomes (67–71). In general, this body of literature suggests that vicarious exposure to a racialized disaster has an effect on birth outcomes and that the effect differs by race/ethnicity (9, 59, 60). For example, Novak et al. (9) demonstrated an increased risk of low birth weight (LBW) among babies born to Latinx people compared to babies born to White people after vicarious exposure to an immigration raid in Postville, IA. Additionally, Lauderdale et al. (60) reported that babies born to Arabic-named people in California had a higher risk of LBW and preterm birth (PTB) after vicarious exposure to the 9/11 attacks in 2001. Finally, two international studies that examined vicarious exposures to two different terrorist attacks (London on 7/7/2005 and the 9/11 attacks in the US) reported decreased birthweights and increased risks of small-for-gestational-age (SmGA) (63, 64). Although not specifically studying race, these international studies provide evidence that vicarious exposure to highly stressful events, likely through widespread media coverage, can affect birth outcomes (63, 64).

The mental and physical health effects of vicarious exposure to racism have also been observed in a small body of literature on collective trauma (72–75). Collective trauma refers to the trauma experienced directly by a population during and after a large-scale disaster in which individuals who are not directly exposed to the disaster identify with individuals directly exposed based on common characteristics (e.g., religion, racial or ethnic group, sex, etc.) (72). After the traumatic circumstances are over (e.g., the Holocaust, hurricanes) the trauma is passed on intergenerationally through oral or written histories (73, 74). It is thus plausible that Black residents of Michigan may vicariously experience symptoms of a collective trauma after observing images via social and traditional media of persons directly exposed to the Flint Water Crisis, if they share a common group status (i.e., racial group) (72, 75).

The Flint Water Crisis is a sustained “event” where Black residents of Michigan may identify with the residents of the city of Flint. Flint is a postindustrial, majority-Black city where over 40% of residents live in poverty (76). Under Michigan's emergency manager law, which targets financially distressed municipalities, the city was subject to state oversight from 2002–2004 and 2011–2015 (77–79). In April 2014, the state-appointed emergency manager switched the city's source of drinking water from the Detroit water system to the Flint River to save money. As an additional cost-cutting measure, officials decided against chemically treating Flint River water which would have prevented corrosion in supply pipes (79–83). While residents voiced complaints about water quality as early as May 2014, and the Flint General Motors plant lodged complaints about Flint's water corroding car parts in October 2014, city leaders failed to take action until September 2015, following the release of reports showing elevated lead levels in the city's water (82) and elevated blood lead levels in children (84). On January 5, 2016, nearly 2 years after the water crisis began, Governor Rick Snyder issued a state of emergency in Flint. Two weeks later, President Barack Obama issued a federal emergency declaration, and local, regional, and national news outlets began to feature stories about the water crisis (85). Many of these stories suggested that systemic biases against Black Americans contributed to the crisis and to the state and federal crisis response (86).

The FWC was the result of longstanding and historic legally sanctioned structural racism that includes “northern style” segregation (i.e., segregation occurring in non-Jim Crow states that relied upon local ordinances, *de facto* property contracts, and federal laws) that was pervasive in nearly every facet of life in Flint, including housing, employment opportunities, education, and other areas, since Flint became a city (79, 87). Prior to the Fair Housing Act of 1968, Black people were severely limited in where they could live in US towns and cities, including Flint. The race-based housing restrictions were legal through both Supreme Court rulings and federal legislation that enabled “redlining,” or color coding areas on a map in red, where banks *assumed* that the residents would have difficulty repaying the federally backed mortgage loan (79, 88–91). Federal segregation policies resulted in many resource deprived and deteriorating cities (79, 89).

The consequences of federal policies that enabled redlining are at the very core of the demographic and economic make-up of modern-day Flint. In its early days, the city of Flint flourished with a strong tax base of mostly White residents and substantial employment opportunities (79, 87). Flint was also highly segregated, initially permitting Black residents to reside in only one corner of the city (79, 87). As jobs became available for all skill-levels, the Black community quickly became overcrowded (79, 87). With a population of about 100,000 after “white flight” (White residents leave when non-White people move into a predominantly White neighborhood) (92) in the 1960s, the demographics shifted to a slightly majority Black and economically disadvantaged community (79). This shift in finances and a strengthening of the Michigan emergency management laws in 2011 enabled the Michigan governor to appoint an unelected city manager with accountability only to the governor (77, 79, 87, 93). Flint's history and struggle with structural racism is why and how the Flint Water Crisis occurred (79). Indeed, this humanmade environmental disaster went virtually unnoticed by the national media for over a year despite substantial complaints from Flint residents, local government, major employers, and researchers (79).

While residents of Flint were directly harmed by the water crisis, the idea that the crisis was rooted in racism likely reverberated in Black communities throughout the state and country (94–96). Vicarious exposure to the FWC includes the substantial increase in social and traditional media coverage when then President Barack Obama (US), Governor Rick Snyder (Michigan), and Mayor Karen Weaver (Flint) declared a state of emergency in Flint in early January 2016 (85). While the Michigan Civil Rights Commission defined the FWC as the result of structural racism, and despite national attention raised by the #BlackLivesMatter movement (97), much of the media neglected to tell the story about the FWC (86). In fact, the increased media attention occurred about 3 months *after* the contaminated water was switched back to the treated water via the Detroit/Lake Huron system. To the extent that pregnant Black people in Michigan attributed the FWC to structural racism and perceived the environmental crisis as stressful, as those residing in Flint did (98), indirect or vicarious exposure to the water crisis may have adversely affected their health and the health of their offspring. Several complex immune, inflammatory, and endocrine pathways are proposed to link psychosocial stressors and birth outcomes (99). Stress can exacerbate conditions such as pre-eclampsia that may lead to early labor, resulting in shorter gestational age (GA) or lower birthweight (BW) (100–104). Stress can also result in restricted fetal growth, which may lead to lower BW or smaller size-for-gestational-age (SzGA) (100).

We hypothesize that witnessing the effects of structural racism on members of their own racial group in Flint, a majority-Black community, negatively affected the psychological well-being of pregnant Black people in Michigan communities outside of Flint, which, in turn, negatively affected the health of their offspring via stress-related mechanisms (20, 105). Drawing on previous research documenting the consequences of vicarious exposure to racialized stressors on the health of historically marginalized groups (9–11, 60, 106), we expect to find decreased BW, GA, and SzGA for babies born to Black people in Michigan communities outside of Flint, following the governor's highly-publicized January 2016 emergency declaration in Flint in comparison to the same time period between 2013 and 2015. We expect to find no changes in birth outcomes for babies born to White people.

## Materials and methods

### Data

We obtained data from the Michigan Department of Health and Human Services (MDHHS) for all singleton live births between 1/1/2013 and 12/31/2016 (*n* = 437,713). The analytic sample includes Non-Hispanic Black (hereafter Black) and Non-Hispanic White (hereafter White) birthing parent-infant pairs living outside the city of Flint. For people with multiple pregnancies across the study period, we used simple random sampling to select one pregnancy per parent. The final analytic sample includes 226,672 births (Supplementary Figure 1).

### Measurement

#### Outcomes

Primary outcomes included BW in grams, GA in weeks, and SzGA z-score. BW and GA were recorded on the standard birth certificate for each birth. SzGA z-scores were computed by comparing BW and GA to the 2010 distribution of SzGA in the standard US population (107).

#### Exposure

Infants were considered exposed to the FWC emergency declaration if they were born in the 37-week period following the governor's emergency declaration (1/5/2016-9/30/2016) and unexposed if they were born during the same 37-week period in 2013, 2014, or 2015, which allows for the comparison across the same time period in birth outcomes (108). The January-September 2016 time period was selected because it coincided with the substantial media attention that occurred as a result of federal, state, and local leaders declaring a state of emergency in Flint (85). We chose 37 weeks because it is the earliest week of gestation defined as “early term” (109).

#### Effect modifier

Race of the birthing parent is recorded on the standard birth certificate. We restricted to Non-Hispanic Black and Non-Hispanic White people, excluding people from other racial and ethnic groups as well as multiracial people.

#### Covariates

Covariates included risk factors for adverse birth outcomes: birthing parent age, education, marital status, source of payment (i.e., private, Medicaid, self-pay, and other), receipt of WIC, pre-pregnancy body mass index (BMI), parity, and infant sex, all of which were provided on the standard birth certificate. Geographic region was derived from location variables provided by MDHHS based on the home address of the birthing parent. We combined minor civil divisions (e.g., cities, townships, and villages) and counties to categorize ten geographic regions by Michigan Prosperity Zones/Regions, which are state defined geographies (110). Potential mediators, which are examined in sensitivity analyses, include the Kessner Adequacy of Prenatal Care Index (111), smoking during pregnancy, alcohol use during pregnancy, gestational diabetes, pre-existing diabetes and/or pre-existing/gestational hypertension, previous preterm birth (PTB) or other poor outcome, and sexually transmitted infections (STIs). Both individual-level covariates and hypothesized mediators are available on the standard birth certificate.

### Data management

We used Basso's data cleaning algorithm to exclude births with implausible or out-of-range GA and BW (112). The analytic sample includes births occurring between January 5th and September 30th each year and excludes births missing place of residence information (*n* = 226,672; Supplementary Figure 1).

The missingness for covariates ranged from 0 to 3.78% (Table 1). To address missingness, we performed 10 multiple imputations. All variables of interest, including the three outcome variables, were included in the imputation model, according to best practice (113). We multiply imputed the missing variables using the fully conditional specification (FCS) method, or imputation by chained equations. We used the mice package in R for imputation (114).

**Table 1:** Descriptive statistics by birthing parent race and exposure to the Flint Water Crisis emergency declaration in Michigan (*n* = 226,672).

	**Percent missing (%)**	**Total** ***n* = 226,672**	**Non-Hispanic Black** ***n =* 45,613**	**Non-Hispanic White** ***n =* 181,059**	***P***	**Unexposed *n =* 171,328**	**Exposed** ***n =* 55,344**	***P***
**Birthing parent race**, ***n*** **(%)**	0.00							
Non-Hispanic Black		45,613 (20.1)				34,540 (20.2)	11,073 (20.0)	0.4364
Non-Hispanic White		181,059 (79.9)				136,788 (79.8)	44,271 (80.0)	
**Birthing parent age, mean (SD)**	0.00	28.13 (5.66)	26.18 (5.93)	28.62 (5.48)	**<0.0001**	28.08 (5.69)	28.27 (5.58)	**<0.0001**
**Birthing parent education**, ***n*** **(%)**	0.62							
Less than high school		23,074 (10.2)	7,916 (17.4)	15,158 (8.4)	**<0.0001**	17,786 (10.4)	5,288 (9.6)	**<0.0001**
High school/GED		56,426 (24.9)	16,120 (35.3)	40,306 (22.3)		42,712 (24.9)	13,714 (24.8)	
Some college		58,811 (25.9)	14,067 (30.8)	44,744 (24.7)		45,031 (26.3)	13,780 (24.9)	
College or more		86,958 (38.4)	6,897 (15.1)	80,061 (44.2)		64,897 (37.9)	22,061 (39.9)	
**Birthing parent marital status**, ***n*** **(%)**	0.03							
Never married		90,073 (39.7)	35,626 (78.1)	54,447 (30.1)	**<0.0001**	68,116 (39.8)	21,957 (39.7)	**0.9234**
Married		129,535 (57.1)	9,194 (20.2)	120,341 (66.5)		97,869 (57.1)	31,666 (57.2)	
Divorced/Widowed		7,005 (3.1)	773 (1.7)	6,232 (3.4)		5,300 (3.1)	1,705 (3.1)	
**Source of payment for delivery**, ***n*** **(%)**	0.36							
Private insurance		127,197 (56.1)	15,941 (34.9)	111,256 (61.4)	**<0.0001**	95,814 (55.9)	31,383 (56.7)	**<0.0001**
Medicaid		93,369 (41.2)	28,001 (61.4)	65,368 (36.1)		70,660 (41.2)	22,709 (41.0)	
Self-Pay		3,198 (1.4)	485 (1.1)	2,713 (1.5)		1,788 (1.0)	298 (0.5)	
Other		2,086 (0.9)	1,069 (2.3)	1,017 (0.6)		2,379 (1.4)	819 (1.5)	
**Receipt of WIC during pregnancy**, ***n*** **(%)**	1.46							
Yes		90,749 (40.0)	29,871 (65.5)	60,878 (33.6)	**<0.0001**	70,363 (41.1)	20,386 (36.8)	**<0.0001**
No		132,620 (58.5)	14,923 (32.7)	117,697 (65.0)		98,396 (57.4)	34,224 (61.8)	
**Pre-pregnancy BMI, mean (SD)**	3.78	27.22 (6.91)	28.88 (7.73)	26.82 (6.64)	**<0.0001**	27.18 (6.90)	27.35 (6.95)	**<0.0001**
**Parity (including birth on record), mean (SD)**	0.03	2.49 (1.70)	2.83 (1.20)	2.41 (1.61)	**<0.0001**	2.52 (1.70)	2.42 (1.70)	**<0.0001**
**Infant Sex**, ***n*** **(%)**	0.00							
Female		110,644 (48.8)	22,592 (49.5)	88,052 (48.6)	**0.0006**	83,698 (48.9)	26,946 (48.7)	0.5014
Male		116,028 (51.2)	23,021 (50.5)	93,007 (51.4)		87,630 (51.1)	28,398 (51.3)	
**Residential geographic region**, ***n*** **(%)**	0.00							
Upper peninsula prosperity alliance		5,689 (2.5)	25 (0.1)	5,664 (3.1)	**<0.0001**	4,353 (2.5)	1,336 (2.4)	0.1505
Northwest prosperity region		6,363 (2.8)	36 (0.1)	6,327 (3.5)		4,777 (2.8)	1,586 (2.9)	
Northeast prosperity region		3,800 (1.7)	19 (0.0)	3,781 (2.1)		2,896 (1.7)	904 (1.6)	
West Michigan prosperity alliance		37,843 (16.7)	3,753 (8.2)	34,090 (18.8)		28,649 (16.7)	9,194 (16.6)	
East Central Michigan prosperity Region		12,113 (5.3)	1,481 (3.2)	10,632 (5.9)		9,194 (5.4)	2,919 (5.3)	
East Michigan prosperity region		17,133 (7.6)	1,345 (2.9)	15,788 (8.7)		12,956 (7.6)	4,177 (7.5)	
South Central prosperity region		10,381 (4.6)	1,537 (3.4)	8,844 (4.9)		7,779 (4.5)	2,602 (4.7)	
Southwest prosperity region		19,023 (8.4)	2,952 (6.5)	16,071 (8.9)		14,480 (8.5)	4,543 (8.2)	
Southeast Michigan prosperity region		21,567 (9.5)	1,895 (4.2)	19,672 (10.9)		16,332 (9.5)	5,234 (9.5)	
Detroit Metro prosperity region		92,760 (40.9)	32,570 (71.4)	60,190 (33.2)		69,911 (40.8)	22,849 (41.3)	
**Birthweight in grams, mean (SD)**	0.00	3331.6 (571.1)	3110.8 (603.9)	3387.2 (548.7)	**<0.0001**	3335.7 (569.4)	3319.0 (576.2)	**<0.0001**
**Gestational age in weeks, mean (SD)**	0.00	38.76 (2.00)	38.33 (2.44)	38.87 (1.86)	**<0.0001**	38.76 (1.99)	38.75 (2.04)	0.4230
**Size for gestational Age Z-score, mean (SD)**	0.00	0.05 (1.03)	−0.29 (0.99)	0.14 (1.02)	**<0.0001**	0.06 (1.03)	0.03 (1.0)	**<0.0001**

Percent missing is denoted as the percentage of observation that are multiply imputed (10 imputed data sets).

Bolded P-values denote statistical significance at α = 0.05. SD = standard deviation.

### Statistical analysis

First, we used non-imputed data to calculate descriptive statistics, including frequencies and percentages for categorical variables and means and standard deviations for continuous variables. Next, we used linear regression models with imputed data to assess the unadjusted association between exposure to the FWC emergency declaration and BW, GA, and SzGA z-score (Model 1). In Model 2, we examined unadjusted associations of race with BW, GA, and SzGA z-score. We then examined associations of each of the birth outcomes with exposure to the FWC emergency declaration and race in covariate-adjusted models (Model 3). Finally, we examined an interaction between exposure to the FWC emergency declaration and race on BW, GA, and SzGA z-score in the model adjusted for covariates (Model 4). Model 4 takes the following form for each outcome:


$$\begin{array}{l} Y_{\text{Outcome}}\;=\;{\text{β}}_{\text{o}}\;+\;{\text{β}}_{1}race\;+\;{\text{β}}_{2}exposure\;+\;{\text{β}}_{3}(race*exposure)\\ \;+\;{\text{β}}_{\text{j}}covariates,j=1\text{ to }9 \end{array}$$

For all models, we report beta coefficients, standard errors, and p-values. We also present predicted marginal means with 95% Wald-type confidence intervals (95% CI) for BW, GA, and SzGA z-score by birthing parent race and exposure status, along with least-square (LS) mean differences (95% CI) between exposed and unexposed infants within race. Statistical significance was assessed at the alpha = 0.05 significance level. Analyses were conducted in SAS 9.4 (115).

#### Sensitivity analysis

We performed several sensitivity analyses, which are displayed in Supplementary materials. First, we examined whether results are sensitive to adjustment for potential mediators. Second, we used logistic regression models to examine the prevalence of dichotomized versions of the birth outcomes (low birthweight, preterm birth, and small-for-gestational-age). Third, we disaggregated unexposed births by year and examined three separate models comparing births in 2013 (before the FWC began), 2014 (after the FWC began but before widespread media coverage), and 2015 (after the FWC began but before widespread media coverage) to births in 2016 (after the emergency declaration). Fourth, we examined a single model in which unexposed births were disaggregated by year to observe any trends in the outcomes over time. Fifth, we stratified models by trimester of exposure to examine potential sensitive periods for exposure to the stressor of the FWC emergency declaration. Sixth, we examined models excluding all births in Genesee County, the county that contains the city of Flint, since people who lived in close proximity to the city may have been exposed to the contaminated drinking water due to their employment in Flint. Finally, we examined an alternative approach to defining exposure to the water crisis. In the last sensitivity analysis, infants were considered exposed if they were born in the 12-month period from September 1, 2015 to August 31, 2016 and unexposed if they were born before September 1, 2015. The alternative start date was selected because it coincides with a small increase in mainly local and regional media attention after a local pediatrician reported elevated blood-lead levels in Flint children (84, 85). A 1-year period was selected to take into account media coverage that continued after the January emergency declaration.

## Results

Table 1 displays descriptive statistics by race and exposure to the FWC emergency declaration. Of 226,672 births, 45,613 infants were born to Black people (20.1%) and 55,344 infants were born after the emergency declaration (24.4%). There were statistically significant Black/White differences in most covariates. Based on birth certificate data, Black parents were younger, less likely to have attained a college education, while being more likely to use Medicaid insurance and receive WIC. Black people were less likely to be married and more likely to live in the Detroit Metro Prosperity Region. The percent of female births was higher among babies born to Black people. There were no statistically significant differences by exposure to the FWC emergency declaration in infant sex, birthing parent race, marital status, or region of residence. Birthing parent age, educational level, private insurance use, and pre-pregnancy BMI were higher among the group exposed to the FWC emergency declaration, while receipt of WIC and parity were lower. Though statistically significant, differences in covariates by exposure status were small in magnitude compared to differences in covariates by race. Descriptive statistics for hypothesized mediators are found in Supplementary Table 1. Black people were more likely to have inadequate prenatal care and an STI during pregnancy, while White people were more likely to use tobacco and have gestational diabetes during pregnancy.

Table 2 displays linear regression coefficients, standard errors (SE), and *P*-values for the unadjusted and adjusted models for BW. In unadjusted models, infants born in the 37 weeks following the governor's emergency declaration had lower BW than infants born during the same 37-week period of the previous three years (b = −16.65, *P* < 0.0001; Model 1), and infants born to Black people had lower BW than infants born to White people (b = −276.41, *P* < 0.0001; Model 2). After adjustment of covariates in Model 3, the beta coefficient for exposure to the FWC emergency declaration increased in magnitude, becoming more negative (b = −18.72, *P* < 0.0001), while the beta coefficient for birthing parent race was attenuated (b = −224.57, *P* < 0.0001). As shown in Model 4 of Table 2, the interaction between exposure to the FWC emergency declaration and birthing parent race was not statistically significant (b = −10.93, *P* = 0.1035).

**Table 2:** Linear regression coefficients for regression of birthweight (grams) on exposure to the Flint Water Crisis emergency declaration in Michigan (*n* = 226,672).

	**Model 1**			**Model 2**			**Model 3**			**Model 4**		
**Variable**	**Beta**	**SE**	***P***	**Beta**	**SE**	***P***	**Beta**	**SE**	***P***	**Beta**	**SE**	***P***
Intercept	3335.68	1.38	**<0.0001**	3387.23	1.32	**<0.0001**	3249.84	9.30	**<0.0001**	3249.19	9.30	**<0.0001**
**Exposed** ^a^												
Yes	−16.65	2.79	**<0.0001**				−18.72	2.69	**<0.0001**	−16.53	3.00	**<0.0001**
No	Ref						Ref			Ref		
**Birthing parent race**												
Non-Hispanic Black				−276.41	2.94	**<0.0001**	−224.57	3.40	**<0.0001**	−221.89	3.77	**<0.0001**
Non-Hispanic White				Ref			Ref			Ref		
**Birthing parent age**							−4.08	0.28	**<0.0001**	−4.08	0.28	**<0.0001**
**Birthing parent education**												
< High school							−153.73	4.88	**<0.0001**	−153.82	4.87	**<0.0001**
High school/GED							−94.31	3.60	**<0.0001**	−94.29	5.60	**<0.0001**
Some college							−45.83	3.83	**<0.0001**	−45.83	3.29	**<0.0001**
College or more							Ref			Ref		
**Birthing parent marital status**												
Never married							−67.02	3.14	**<0.0001**	−67.05	3.14	**<0.0001**
Married							Ref			Ref		
Divorced/widowed							−94.87	6.90	**<0.0001**	−94.89	6.90	**<0.0001**
**Source of payment for delivery**												
Private insurance							Ref			Ref		
Medicaid							−56.39	3.11	**<0.0001**	−56.24	3.11	**<0.0001**
Self-Pay							44.76	9.84	**<0.0001**	44.78	9.84	**<0.0001**
Other							−31.97	12.28	**0.0092**	−32.69	12.28	**0.0078**
**Receipt of WIC during pregnancy**												
Yes							0.47	3.13	0.8813	0.40	3.13	0.8979
No							Ref			Ref		
**Pre-pregnancy BMI**							8.86	0.18	**<0.0001**	8.86	0.18	**<0.0001**
**Parity (including birth on record)**							13.60	0.79	**<0.0001**	13.60	0.79	**<0.0001**
**Infant's sex**												
Female							Ref			Ref		
Male							120.77	2.31	**<0.0001**	120.77	2.30	**<0.0001**
**Residential geographic region**												
Upper peninsula prosperity alliance							40.88	7.60	**<0.0001**	40.91	7.60	**<0.0001**
Northwest prosperity region							54.72	7.21	**<0.0001**	54.70	7.21	**<0.0001**
Northeast prosperity region							24.42	9.19	**0.0079**	24.44	9.19	**0.0078**
West Michigan prosperity alliance							30.89	3.46	**<0.0001**	30.90	3.46	**<0.0001**
East Central Michigan prosperity region							16.07	5.38	**0.0028**	16.08	5.38	**0.0028**
East Michigan prosperity region							5.39	4.67	0.2486	5.40	4.67	0.2476
South Central prosperity region							15.09	5.72	**0.0084**	15.07	5.72	**0.0084**
Southwest prosperity region							19.77	4.44	**<0.0001**	19.78	4.44	**<0.0001**
Southeast Michigan prosperity region							16.41	4.23	**0.0001**	16.43	4.23	**0.0001**
**Detroit Metro prosperity region**							**Ref**			**Ref**		
**Interaction**												
Race*Exposure										−10.93	6.71	0.1035

a Exposed infants were born between 1/5/2016 and 9/30/2016; unexposed infants were born in the same 37-week period in 2013, 2014, or 2015. Bolded *P*-values denote statistical significance at α = 0.05. Sample Sizes Non-Hispanic Black: 34,540 (unexposed), 11,073 (exposed); Non-Hispanic White: 136,788 (unexposed), 44,271 (exposed).

Table 3 displays the unadjusted and adjusted linear regression coefficients for GA. In unadjusted models, there was no difference in GA between people exposed and unexposed to the FWC emergency declaration (b = −0.008, *P* = 0.4258; Model 1), while infants born to Black people had lower GA compared to infants born to White people (b = −0.55, *P* < 0.0001; Model 2). After adjusting for covariates in Model 3, the beta coefficients for exposure to the FWC emergency declaration (b = −0.007, *P* = 0.4780) and birthing parent race (b = −0.44, *P* < 0.0001) were attenuated. As shown in Model 4 of Table 3, the interaction between exposure to the FWC declaration and race was statistically significant (b = −0.06, *P* = 0.0216).

**Table 3:** Linear regression coefficients for regression of gestational age (weeks) on exposure to the Flint Water Crisis emergency declaration in Michigan (*n* = 226,672).

	**Model 1**			**Model 2**			**Model 3**			**Model 4**		
**Variable**	**Beta**	**SE**	***P***	**Beta**	**SE**	***P***	**Beta**	**SE**	***P***	**Beta**	**SE**	***P***
**Intercept**	38.76	0.005	**<0.0001**	39.87	0.005	**<0.0001**	39.84	0.03	**<0.0001**	39.84	0.03	**<0.0001**
**Exposed** ^a^												
Yes	−0.008	0.01	0.4258				−0.007	0.01	0.4780	0.004	0.01	0.6962
No	Ref						Ref			Ref		
**Birthing parent race**												
Non-Hispanic Black				−0.55	0.01	**<0.0001**	−0.44	0.01	**<0.0001**	−0.42	0.01	**<0.0001**
Non-Hispanic White				Ref			Ref			Ref		
**Birthing parent age**							−0.02	0.001	**<0.0001**	−0.02	0.001	**<0.0001**
**Birthing parent education**												
< High school							−0.29	0.02	**<0.0001**	−0.30	0.02	**<0.0001**
High school/GED							−0.21	0.01	**<0.0001**	−0.21	0.01	**<0.0001**
Some college							−0.12	0.01	**<0.0001**	−0.12	0.01	**<0.0001**
College or more							Ref			Ref		
**Birthing parent marital status**												
Never Married							−0.14	0.01	**<0.0001**	−0.14	0.01	**<0.0001**
Married							Ref			Ref		
Divorced/Widowed							−0.25	0.02	**<0.0001**	−0.25	0.02	**<0.0001**
**Source of payment for delivery**												
Private insurance							Ref			Ref		
Medicaid							−0.10	0.01	**<0.0001**	−0.09	0.01	**<0.0001**
Self-Pay							0.23	0.04	**<0.0001**	0.23	0.04	**<0.0001**
Other							−0.05	0.04	0.7320	−0.02	0.04	0.6711
**Receipt of WIC during pregnancy**												
Yes							0.12	0.01	**<0.0001**	0.12	0.01	**<0.0001**
No							Ref			Ref		
**Pre-pregnancy BMI**							−0.002	0.001	**0.0005**	−0.002	0.001	**0.0005**
**Parity (including birth on record)**							−0.05	0.003	**<0.0001**	−0.05	0.003	**<0.0001**
**Infant's sex**												
Female							−0.10	0.01	**<0.0001**	−0.09	0.01	**<0.0001**
Male							Ref			Ref		
**Residential geographic region**												
Upper peninsula prosperity alliance							0.13	0.03	**<0.0001**	0.13	0.03	**<0.0001**
Northwest prosperity region							0.22	0.03	**<0.0001**	0.22	0.03	**<0.0001**
Northeast prosperity region							0.12	0.03	**0.0002**	0.12	0.03	**0.0002**
West Michigan prosperity alliance							0.03	0.01	**0.0130**	0.03	0.01	**0.0129**
East Central Michigan prosperity region							0.11	0.02	**<0.0001**	0.11	0.02	**<0.0001**
East Michigan prosperity region							−0.07	0.02	**<0.0001**	−0.07	0.02	**<0.0001**
South Central Prosperity Region							0.08	0.02	**0.0001**	0.08	0.02	**0.0001**
Southwest prosperity region							0.17	0.02	**<0.0001**	0.17	0.02	**<0.0001**
Southeast Michigan prosperity region							0.09	0.02	**<0.0001**	0.09	0.02	**<0.0001**
Detroit Metro prosperity region							Ref			Ref		
**Interaction**												
Race*Exposure										−0.06	0.02	**0.0216**

a Exposed infants were born between 1/5/2016 and 9/30/2016; unexposed infants were born in the same 37-week period in 2013, 2014, or 2015.

Bolded *P*-values denote statistical significance at α = 0.05.

Sample Sizes Non-Hispanic Black: 34,540 (unexposed), 11,073 (exposed); Non-Hispanic White: 136,788 (unexposed), 44,271 (exposed).

Table 4 displays the unadjusted and adjusted linear regression coefficients for SzGA z-score. In unadjusted models, infants born in the 37 weeks after the FWC emergency declaration had significantly lower SzGA z-score than infants born in the same 37-weeks in the previous 3 years (b = −0.03, *P* < 0.0001; Model1), and infants born to Black people had significantly lower SzGA z-score compared to infants born to White people (b = −0.43, *P* < 0.0001; Model 2). After adjusting for covariates in Model 3, the beta coefficient for exposure increased in magnitude, becoming more negative (b = −0.04, *P* < 0.0001) while the beta coefficient for race was attenuated (b = −0.35, *P* < 0.0001). As shown in Model 4, the interaction between exposure to the FWC emergency declaration and birthing parent race was not statistically significant (b = −0.01, *P* = 0.6562).

**Table 4:** Linear regression coefficients for regression of size for gestational age (z-score) on exposure to the Flint Water Crisis emergency declaration in Michigan (*n* = 226,672).

	**Model 1**			**Model 2**			**Model 3**			**Model 4**		
**Variable**	**Beta**	**SE**	***P***	**Beta**	**SE**	***P***	**Beta**	**SE**	***P***	**Beta**	**SE**	***P***
**Intercept**	0.06	0.002	**<0.0001**	0.14	0.002	**<0.0001**	−0.37	0.02	**<0.0001**	−0.37	0.02	**<0.0001**
**Exposed** ^a^												
Yes	−0.03	0.01	**<0.0001**				−0.04	0.005	**<0.0001**	−0.04	0.01	**<0.0001**
No	Ref						Ref			Ref		
**Birthing parent race**												
Non-Hispanic Black				−0.43	0.005	**<0.0001**	−0.35	0.006	**<0.0001**	−0.35	0.01	**<0.0001**
Non-Hispanic White							Ref			Ref		
**Exposed** ^**a**^												
Yes							−0.04	0.005	**<0.0001**	−0.04	0.01	**<0.0001**
No							Ref			Ref		
**Birthing parent age**							−0.002	0.001	**0.0021**	−0.002	0.001	**0.0021**
**Maternal education**												
< High school							−0.24	0.01	**<0.0001**	−0.24	0.01	**<0.0001**
High school/GED							−0.14	0.01	**<0.0001**	−0.14	0.01	**<0.0001**
Some college							−0.06	0.01	**<0.0001**	−0.06	0.01	**<0.0001**
College or more							Ref			Ref		
**Birthing parent marital status**												
Never married							−0.09	0.01	**<0.0001**	−0.09	0.01	**<0.0001**
Married							Ref			Ref		
Divorced/widowed							−0.12	0.01	**<0.0001**	−0.12	0.01	**<0.0001**
**Source of payment for delivery**												
Private insurance							Ref			Ref		
Medicaid							−0.09	0.01	**<0.0001**	−0.09	0.01	**<0.0001**
Self-Pay							0.07	0.02	**<0.0001**	0.07	0.02	**<0.0001**
Other							−0.06	0.02	**0.0050**	−0.06	0.02	**0.0048**
**Receipt of WIC during pregnancy**												
Yes							−0.06	0.01	**<0.0001**	−0.06	0.01	**<0.0001**
No										Ref		
**Pre-pregnancy BMI**							0.02	0.0003	**<0.0001**	0.02	0.0003	**<0.0001**
**Parity (including birth on record)**							0.04	0.001	**<0.0001**	0.04	0.001	**<0.0001**
**Infant's sex**												
Female							0.02	0.004	**<0.0001**	0.02	0.004	**<0.0001**
Male							Ref			Ref		
**Residential geographic region**												
Upper Peninsula prosperity alliance							0.06	0.01	**<0.0001**	0.06	0.01	**<0.0001**
Northwest prosperity region							0.07	0.01	**<0.0001**	0.07	0.01	**<0.0001**
Northeast prosperity region							0.02	0.02	0.1823	0.02	0.02	0.1821
West Michigan prosperity alliance							0.06	0.01	**<0.0001**	0.06	0.01	**<0.0001**
East Central Michigan prosperity region							0.00	0.01	0.6360	0.005	0.01	0.6354
East Michigan prosperity region							0.03	0.01	**0.0001**	0.03	0.01	**0.0001**
South Central prosperity region							0.02	0.01	0.0813	0.02	0.01	0.0814
Southwest prosperity region							0.00	0.01	0.9873	0.0001	0.01	0.9872
Southeast Michigan prosperity region							0.01	0.01	0.1383	0.01	0.01	0.1380
Detroit metro prosperity region							Ref			Ref		
**Interactions**												
Race*Exposure										−0.01	0.01	0.6562

a Exposed infants were born between 1/5/2016 and 9/30/2016; unexposed infants were born in the same 37-week period in 2013, 2014, or 2015.

Bolded *P*-values denote statistical significance at α = 0.05.

Sample Sizes Non-Hispanic Black: 34,540 (unexposed), 11,073 (exposed); Non-Hispanic White: 136,788 (unexposed), 44,271 (exposed).

**Figure 1:**
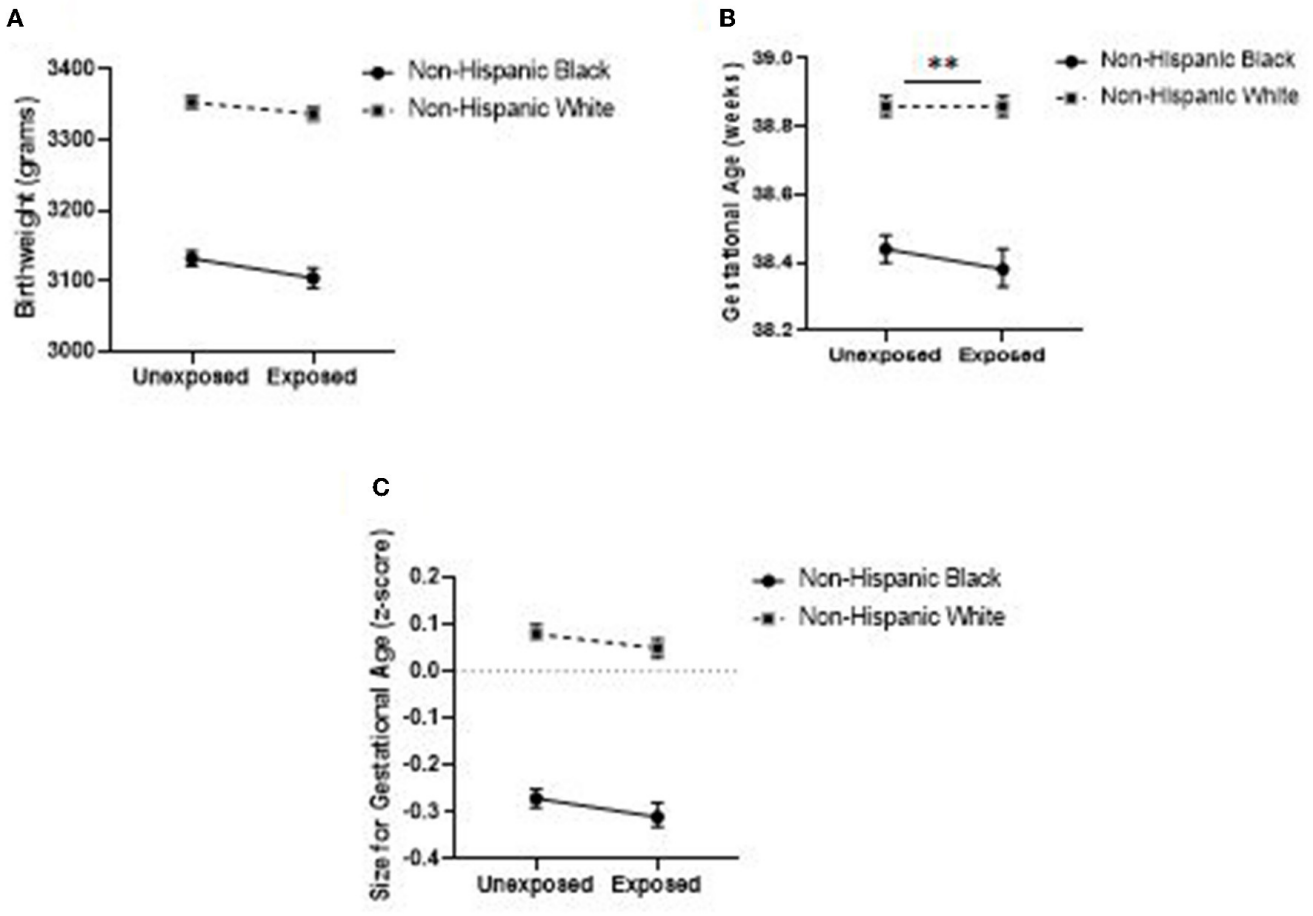
Adjusted means for birthweight **(A)**, gestational age **(B)**, and size-for-gestational-age **(C)** by maternal race and exposure to the Flint Water Crisis emergency declaration in Michigan (*n* = 226,672). Asterisks (**) denote statistical significance of the race-by-exposure interaction at α = 0.05. Means are from fully adjusted models. .

Table 5 displays the predicted means for BW, GA, and SzGA z-score by birthing parent race and exposure to the FWC emergency declaration, as well as LS mean differences between exposed and unexposed infants within race. The estimated mean BW for babies born in the 37-week period after the FWC emergency declaration vs. the same 37-week period in the previous 3 years was 27.5 grams lower (95% CI: −39.2, −15.7; *P* < 0.0001) among babies born to Black people and 16.5 grams lower (95% CI: −22.4, −10.6; *P* < 0.0001) among babies born to White people. Estimated GA was 0.05 weeks lower among exposed vs. unexposed infants born to Black people (95% CI: −0.09, −0.01; *P* = 0.0177), while there was no change in GA among infants born to White people (95% CI: −0.01, 0.03; *P* = 0.6962) following the emergency declaration. Finally, estimated mean SzGA z-score was 0.04 units lower after the emergency declaration among babies born to both Black (95% CI: −0.06, −0.02; *P* = 0.0002) and White (95% CI: −0.05, −0.02; *P* < 0.0001) people. The relationship between exposure to the emergency declaration and GA differed by race, but not for BW or SzGA z-score (see interaction effects in Tables 2–4, Model 4). Figure 1 provides a graphical depiction of results from the fully adjusted models.

**Table 5:** Predicted means^a^ and mean difference for birth outcomes on exposure to the Flint Water Crisis emergency declaration in Michigan (*n* = 226,672).

	**Birthweight (Grams)**		**Gestational age (Weeks)**		**Size for gestational age (Z-score)**	
	**Non-Hispanic Black**	**Non-Hispanic White**	**Non-Hispanic Black**	**Non-Hispanic White**	**Non-Hispanic Black**	**Non-Hispanic White**
**Exposure status^b^**	**Mean (95% CI)**	**Mean (95% CI)**	**Mean (95% CI)**	**Mean (95% CI)**	**Mean (95% CI)**	**Mean (95% CI)**
Exposed	3104.6	3337.4	38.38	38.86	−0.31	0.05
	(3090.5, 3118.7)	(3327.3, 3347.5)	(38.33, 38.44)	(38.83, 38.90)	(−0.33, −0.28)	(0.03, 0.07)
Unexposed	3132.0	3353.9	38.44	38.86	−0.27	0.08
	(3121.0, 3143.1)	(3344.7, 3363.2)	(38.40, 38.48)	(38.83, 38.89)	(−0.29, −0.25)	(0.07, 0.10)
Least square mean difference	−27.5	−16.5	−0.05	0.0004	−0.04	−0.04
(95% CI)	(−39.2, −15.7)	(−22.4, −10.6)	(−0.09, −0.01)	(−0.01, 0.03)	(−0.06, −0.02)	(−0.05, −0.02)
*P*	**<0.0001**	**<0.0001**	**0.0177**	0.6962	**0.0002**	**<0.0001**

a Linear model (4) stratified by race and exposure status adjusted for covariates: (birthing parent age, education, marital status, source of payment for delivery, receipt of WIC during pregnancy, pre-pregnancy BMI, parity, infant sex, and residential geographic region).

b Exposed infants were born between 1/5/2016 and 9/30/2016; unexposed infants were born in the same 37-week period in 2013, 2014, or 2015.

Bolded *P*-values denote statistical significance at α = 0.05.

Sensitivity analyses including the hypothesized mediators was consistent with the primary analysis. Analyses showed lower BW and SzGA after the emergency declaration for babies born to both Black and White people, with a decrease in GA for babies born to Black people and no change for babies born to White people (Supplementary Table 2).

Sensitivity analyses examining dichotomous outcomes showed higher prevalence of low birthweight (LBW) among babies born to Black and White people after the emergency declaration compared to the previous 3 years, with similar changes for both groups. The prevalence of preterm birth (PTB) did not significantly increase after the emergency declaration for babies born to Black or White people. In the primary analysis for GA, we observed a lower GA, albeit a very small difference (−0.05 weeks, Table 5), for babies born to Black people and no difference for babies born to White people. The prevalence of small-for-gestational-age (SmGA) increased among babies born to Black and White people after the emergency declaration, but the increase was greater for babies born to Black people (Supplementary Table 3).

Additional sensitivity analyses with separate models comparing births in 2013, 2014, and 2015 to births in 2016 produced results similar to the primary analyses and suggest that the statistically significant interaction between race and exposure to the emergency declaration on GA was driven by births occurring in 2013, the year before Flint's emergency manager switched the source of the city's drinking water, and 2014, the early part of water crisis, prior to widespread media coverage (Supplementary Tables 4–6).

We also ran a single model where unexposed births were disaggregated by year (Supplementary Figure 2 and Supplementary Table 7). Similar to the primary analyses, the results of this sensitivity analysis revealed a statistically significant interaction between birthing parent race and exposure to the emergency declaration for GA but not for BW or SzGA. It also revealed a gradual downward trend in GA across each of the years examined for babies born to Black people, rather than a sudden decrease following the emergency declaration.

The next sensitivity analyses examined separate models by trimester of exposure (Supplementary Tables 8–10). First trimester exposure to the FWC emergency declaration was associated with decreased BW and SzGA for babies born to Black and White people, but no change in GA for either group (Supplementary Table 8). Exposure during the second trimester was associated with a greater decrease in BW among babies born to Black people than White people, no change in GA for babies born to Black or White people, and a decrease in SzGA z-score that was similar in magnitude for babies born to Black and White people (Supplementary Table 9). Third trimester exposure was associated with decreased BW and SzGA z-score for babies born to Black and White people, as well as decreased GA for babies born to Black people only (Supplementary Table 10). Trimester-stratified models revealed that associations of the emergency declaration with birth outcomes varied according to the timing of exposure, with associations with timing of labor limited to exposure later in pregnancy.

Next, we examined the results of the primary analysis by excluding all births occurring in Genesee county, the county that contains Flint, MI in order to account for potential direct exposure to contaminated water through commuting patterns. The results indicate, like the primary analysis, the race^*^exposure interaction was not statistically significant for BW or SzGA (Supplementary Tables 11, 13) Also like the primary analysis, after adjusting for covariates, there was a statistically significant race^*^exposure interaction (b = −0.06, *P* = 0.0212) for GA (Supplementary Table 12). Unlike the primary analysis (Supplementary Table 14), the difference between exposed and unexposed Non-Hispanic Black birthing parents was no longer statistically significant (difference = −0.05, 95% CI: −0.10, 0.01, *p* = 0.0802).

In the final sensitivity analysis (Supplementary Tables 15–18), we shifted the start date of the exposure period from January 5, 2016 to September 1, 2015, which coincides with a small increase in local and regional media coverage after a local pediatrician reported elevated blood lead levels in Flint children. In addition, we extended the exposure period to a full year after Sept 1, 2015 through August 31, 2016, herein referred to the modified exposure period. Using the modified exposure period, we observed similar results to the primary analysis including the statistically significant race^*^exposure interaction for GA (*b* = −0.05, *p* = 0.0109). Unlike the primary analysis, the difference between exposed and unexposed Non-Hispanic Black birthing parents was nearly statistically significant (difference = −0.04, 95% CI: −0.08, 0.01, *p* = 0.0874) as opposed to the primary analysis which revealed a statistically significant difference for Non-Hispanic Black birthing parents (difference: −0.05, 95% CI: −0.09, −0.01, *p* = 0.0177).

## Discussion

Structural racism is so embedded in American society that its health impacts are hard to quantify. Building on nascent research examining health-related effects of indirect exposure to structural racism (9–13), this study aimed to document the impact of the FWC on racial disparities in birth outcomes in Michigan communities outside of Flint. Media reports suggested that systemic biases against Black Americans played a role in the crisis (86). A February 2017 report from the Michigan Civil Rights Commission substantiated this claim, concluding that racist policies and practices in employment, housing, and education, as well as racially disparate effects of the state's emergency manager law, contributed to the FWC (79). Additionally, local members of the Flint community have attributed the FWC to structural racism (94, 106, 116, 117). Thus, we hypothesized that Black people in Michigan communities outside of Flint also may have attributed the water crisis to racism and that witnessing the effects of structural racism on members of their own racial/ethnic group in Flint, a majority-Black community, negatively affected their well-being and the health of their offspring via stress-related mechanisms (20, 105). To explore this hypothesis, we used individual birth records for all births in Michigan, excluding Flint, from 2013 to 2016 to examine whether BW, GA, and SzGA decreased among babies born to Black people, but not among babies born to White people, following the highly-publicized January 2016 emergency declaration in Flint.

Contrary to expectations, we observed that BW and SzGA were lower for babies born to both Black *and* White people after the governor's emergency declaration compared to the same 37-week period in the previous 3 years. Interactions between race and exposure to the emergency declaration were not statistically significant, suggesting that associations of exposure to the emergency declaration with BW and SzGA were similar for babies born to Black and White people. Although the magnitude of differences in outcomes between exposed and unexposed infants were similar across racial groups, babies born to Black people were significantly smaller in terms of BW and SzGA compared to babies born to White people in both the unexposed and exposed groups. While disparities in these outcomes did not increase following the emergency declaration, they remained alarmingly high.

In contrast to the results for BW and SzGA, there was a statistically significant, albeit very small, race difference in the association between exposure to the emergency declaration and GA. We found that GA was lower among exposed infants born to Black people, while there was no change among infants born to White people following the emergency declaration. This finding was confirmed by a sensitivity analysis after removing births occurring in all of Genesee County, the county that contains the city of Flint. It was also confirmed in a sensitivity analysis where we modified the exposure period to coincide with a small increase in local and regional media coverage beginning in September 2015. This finding is consistent with the hypothesis that the FWC may have had a negative impact on the health of babies born to Black people, while having no impact on babies born to White people, and is in line with some previous research examining effects of racialized stressors on birth outcomes (9, 60), although other studies have reported null findings (67).

Sensitivity analyses with separate models comparing births in 2013, 2014, and 2015 to births in 2016 suggest that the statistically significant race difference in the association between exposure to the FWC emergency declaration and GA was driven by births occurring in 2013, the year before Flint's emergency manager switched the source of the city's drinking water, and 2014, during the early stages of the water crisis. These results are noteworthy, given that 2013 is the only year in which we are certain that our unexposed group had no knowledge of the water problems in Flint. While media coverage of the water crisis was minimal prior to the governor's emergency declaration (85), it is possible that some residents of Michigan were aware of the situation in Flint prior to 2016, particularly if they had personal connections to Flint. To examine this potential misclassification of exposure, we conducted a sensitivity analysis in which we modified the exposure period shifting it earlier to September 2015 and extending it beyond 37-weeks to a full year. This sensitivity analysis revealed consistent results to the primary analysis. However, there is always the potential for misclassification of exposure. In fact, in a recent study conducted 5 years after the emergency declaration in Flint, Kilpatrick et al. (118) found that Black women in Michigan communities outside of Flint were more likely than White women to know someone who was directly affected by the water crisis. To the extent that 2014 and 2015 births were misclassified as unexposed, results would be biased toward the null.

**Figure 2:**
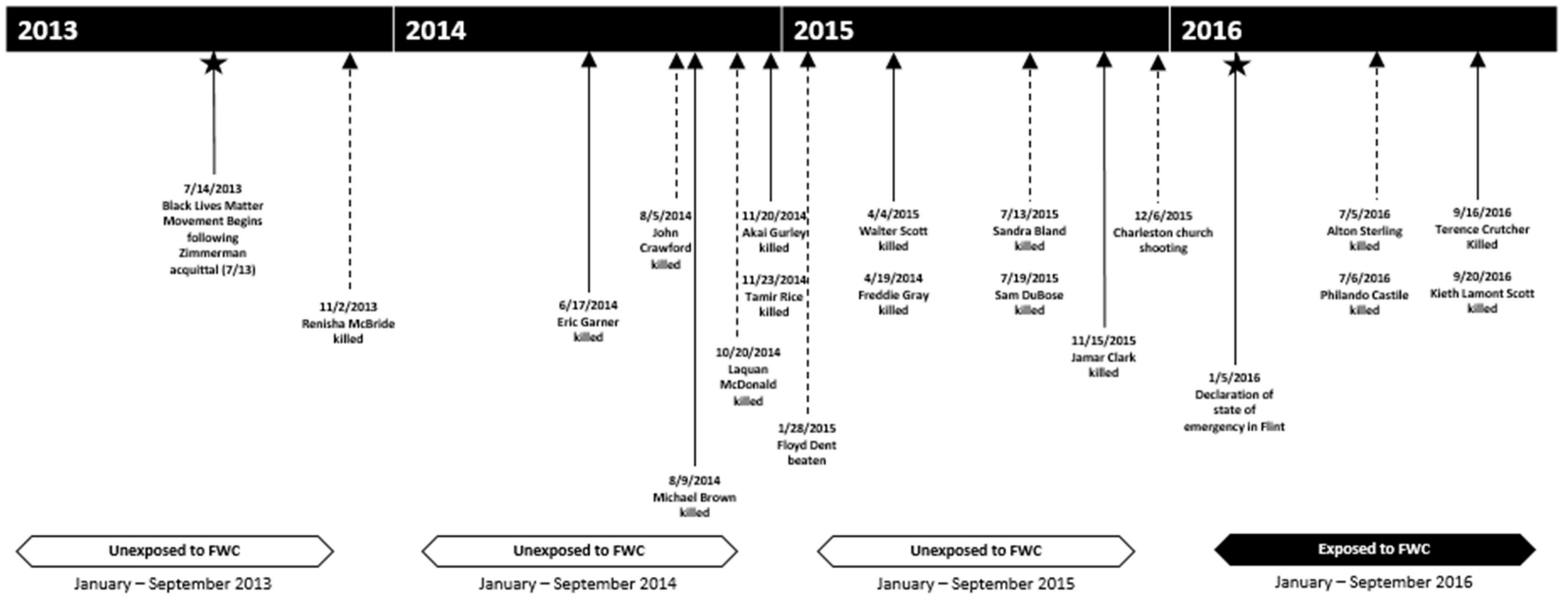
Timeline of highly publicized instances of racialized violence relative to exposure period, 2013–2016.

The sensitivity analysis in which data are disaggregated by year reveals a downward trend for gestational age across each year included in the study, especially for babies born to Black people. This trend is consistent with a nationwide increase in late preterm and early preterm births to Non-Hispanic Black people between 2014 and 2016 (119) and could be attributable to other sources of vicarious structural racism that increased over the same time period, particularly the highly-publicized police-involved deaths of Black people, including Eric Garner, Michael Brown, Laquan McDonald, Tamir Rice, Freddie Gray, and Sandra Bland (Figure 2).

### Strengths and limitations

We examined the FWC as a specific, highly publicized (after the emergency declaration) instance of structural racism, enabling us to quantify the impact of vicarious exposure to structural racism on newborn health. Because we focused on birthing parent and infant pairs who lived outside the city of Flint and were, therefore, not directly exposed to the toxic effects of lead, this work helps to potentially demonstrate that the public health consequences of the water crisis could extend beyond the health-damaging effects of lead exposure on residents of Flint. In fact, we found that exposure to the governor's emergency declaration was associated with lower BW and SzGA for babies born to both Black *and* White people in Michigan, while the association between exposure to the emergency declaration and GA was limited to babies born to Black people.

An additional strength of this study is the focus on birth outcomes. Outcomes like BW, GA, and SzGA are known to be sensitive to maternal stress exposure (102, 120), and the biological mechanisms linking exposure to stress during pregnancy to these outcomes are well-established (100–103, 121, 122). The use of vital statistics was also a strength as it enabled us to capture all births that met our inclusion criteria, minimizing the risk of selection bias. The use of objective outcome data from the birth certificate is another key strength. Prior research indicates that BW data are highly accurate for babies born to Black and White people (123–125). Data on GA are somewhat less accurate, but there is no indication that accuracy varies according to race (126). An additional strength of this study is the analysis of continuous birth outcomes. Associations between indirect exposure to the FWC and birth outcomes are likely to be small and, therefore, may be obscured in an analysis of dichotomous outcomes. Finally, the inclusion of numerous sensitivity analyses suggests that study results are robust to changes in model specification.

Despite these strengths, this study has several limitations. First, the FWC was not a discrete event. It started in 2014, when the emergency manager switched the source of the city's drinking water, but an analysis of traditional media coverage, social media posts, and Google search results suggests that awareness of the water crisis was extremely limited until the governor's emergency declaration in January 2016, even in Michigan (85). While the classification scheme used to define exposed births is supported by the evidence, we acknowledge the potential for exposure misclassification. For example, pregnant people with personal connections to Flint may have been aware of the water crisis long before the emergency declaration (118). To address this limitation, we included births from 2013 in the analysis. Although we are certain that babies born in 2013 were not exposed to news of the water crisis, a 3-year gap between exposed and unexposed births increases the chances of variation in other unmeasured conditions relevant for birth outcomes.

A second limitation is that we cannot fully disentangle the effects of exposure to the FWC from the effects of exposure to other racialized and non-racialized stressors that occurred during the study period (Figure 2). While it is technically possible to adjust for these exposures in our models, the large number of other stressors which were seemingly ubiquitous during the study period, including police involved violence, massive protests to combat police violence, a volatile and racially charged presidential election, White supremacist violence in Charlottesville VA, and/or the Charleston church shooting (racialized stressors), or the threat of Zika infection to pregnant people (non-racialized), renders this approach impractical. Another potential limitation is that residents from Flint may have moved to other areas in Michigan prior to giving birth. In this case, some babies in our study may have been exposed to the contaminated water *in utero*, which would bias results away from the null if exposure to lead and other contaminants increased the risk of adverse birth outcomes as supported by literature on those directly exposed to the FWC (127–129). Women also could have moved out of the state in response to the emergency declaration. Noted earlier, GA is less accurate than other measures as it is a mix of physician and parental report on the birth certificate. We attempted to overcome this limitation by performing a quality control technique based on Basso's algorithm which may have contributed to some measurement error (9, 112). Finally, we do not have information on risk and resilience factors, such as exposure to interpersonal discrimination or social support, which could modify associations of exposure to the FWC with birth outcomes.

## Conclusions

This study suggests that the public health consequences of the FWC may have been more widespread than previously thought. We found that babies born in Michigan communities outside of Flint after the governor's 2016 emergency declaration had worse birth outcomes than babies born during the previous 3 years. Decreases in BW and SzGA were similar for babies born to Black and White people, while a decrease in GA was only observed among babies born to Black people. These results suggest that the water crisis may have raised fears about water contamination for women throughout the state, regardless of race, but that Black people may have perceived the water crisis as more stressful. Future research could focus on the intersectionality of race and resources that might produce further harm (or buffer the harmful effects of racialized stressors) to neonates (e.g., health insurance status, prenatal care access). We have argued that witnessing the effects of an environmental crisis that was widely attributed to structural racism was a unique source of stress for pregnant Black people in Michigan that may have negatively affected their well-being and the health of their offspring. Given increasing media coverage of racialized stressors like the FWC and police killings of unarmed Black people, it is critical that we continue to explore the public health significance of exposure to vicarious structural racism.

## Data availability statement

The data analyzed in this study is subject to the following licenses/restrictions: A data use agreement process was enforced for the acquisition of this birth record data in Michigan, birth record data is publicly available with a data use agreement and a fee. Requests to access these datasets should be directed to https://www.michigan.gov/mdhhs/doing-business/vitalrecords.

## Ethics statement

The studies involving human participants were reviewed and approved by University of Michigan Institutional Review Board; Michigan Department of Health and Human Services Institutional Review Board. Written informed consent from the participants' legal guardian/next of kin was not required to participate in this study in accordance with the national legislation and the institutional requirements.

## Author contributions

KA, BN, and CA: conceptualization. KA, JM, NN, BN, and NF: methodology. KA and BN: data acquisition. JM: data management. KA: original draft preparation and responsible for ensuring that the descriptions are accurate and agreed upon by all authors. KA and JM: visualization. BN: supervision. All authors: reviewing and editing. All authors contributed to the article and approved the submitted version.

## Funding

This work was supported by the National Institute on Minority Health and Health Disparities (Grant R21MD012683) to BN and CA (MPIs).

## Conflict of interest

The authors declare that the research was conducted in the absence of any commercial or financial relationships that could be construed as a potential conflict of interest. The reviewer EC declared a shared affiliation with the authors KA, JM, NF, and BN to the handling editor at the time of review.

## Publisher's note

All claims expressed in this article are solely those of the authors and do not necessarily represent those of their affiliated organizations, or those of the publisher, the editors and the reviewers. Any product that may be evaluated in this article, or claim that may be made by its manufacturer, is not guaranteed or endorsed by the publisher.
