# Supplementary material for: Vicarious structural racism and infant health disparities in Michigan: The Flint Water Crisis
Source: Front Public Health. 2022 Sep 6;10:954896. doi: 10.3389/fpubh.2022.954896 (PMC9486078; doi:10.3389/fpubh.2022.954896)
Supplement: Supplementary file 1 [file Data_Sheet_1.docx]

| **Table S1. Descriptive Statistics by Maternal Race and Exposure to the Flint Water Crisis Emergency Declaration in Michigan for Hypothesized Mediators (n=226,672)** | | | | | | | | |
| --- | --- | --- | --- | --- | --- | --- | --- | --- |
|  | Percent Missing  (%) | Total n=226,672 | Non-Hispanic Black n=45,613 | Non-Hispanic White n=181,059 | *P*-value | Unexposed n=171,328 | Exposed  n=55,344 | *P*-value |
| **Kessner Index, n (%)** | 0.41 |  |  |  |  |  |  |  |
| Adequate |  | 154,832  (68.3) | 25,216  (55.3) | 129,616  (71.6) | **<0.0001** | 116,178  (67.8) | 38,654  (69.8) | **<0.0001** |
| Intermediate |  | 51,428  (22.7) | 13,323  (29.2) | 38,105  (21.0) |  | 39,429  (23.0) | 11,999  (21.7) |  |
| Inadequate |  | 19,472  (8.6) | 6,654  (14.6) | 12,818  (7.1) |  | 14,781  (8.6) | 4,691  (8.5) |  |
| **Tobacco Use during Pregnancy, n (%)** | 0.41 |  |  |  |  |  |  |  |
| Yes |  | 48,140  (21.2) | 7,851  (17.2) | 40,289  (22.3) | **<0.0001** | 37,367  (21.8) | 10,773  (19.5) | **<0.0001** |
| No |  | 177,610  (78.4) | 37,481  (82.2) | 140,129  (77.4) |  | 133,356  (77.8) | 44,254  (80.0) |  |
| **Alcohol Use during Pregnancy, n (%)** | 1.03 |  |  |  |  |  |  |  |
| Yes |  | 1,654  (0.7) | 340  (0.7) | 1,314  (0.7) | 0.7655 | 1,241  (0.7) | 413  (0.7) | 0.5353 |
| No |  | 222,676  (98.2) | 45,115  (98.9) | 177,561  (98.1) |  | 168,536  (98.4) | 54,140  (97.8) |  |
| **Gestational Diabetes, n (%)** | 0.27 |  |  |  |  |  |  |  |
| Yes |  | 10,955  (4.8) | 1,628  (3.6) | 9,327  (5.2) | **<0.0001** | 8,295  (4.8) | 2,660  (4.8) | 0.7037 |
| No |  | 215,104 (94.9) | 43,893  (96.2) | 171,211  (94.6) |  | 162,530  (94.9) | 52,574  (95.0) |  |
| **Pre-Pregnancy Diabetes, Pre-Pregnancy Hypertension and/or Gestational Hypertension, n (%)** | 0.27 |  |  |  |  |  |  |  |
| Yes |  | 17,739  (7.8) | 3,655  (8.0) | 14,084  (7.8) | 0.1058 | 12,723  (7.4) | 5,016  (9.1) | **<0.0001** |
| No |  | 208,320 (91.9) | 41,866  (91.8) | 166,454 (91.9) |  | 158,102  (92.3) | 50,218  (90.7) |  |
| **Previous Preterm Birth and/or Other Previous Poor Pregnancy Outcome, n (%)** | 0.27 |  |  |  |  |  |  |  |
| Yes |  | 9,287  (4.1) | 1,952  (4.3) | 7,335  (4.1) | **0.0305** | 7,467  (4.4) | 1,820  (3.3) | **<0.0001** |
| No |  | 216,767 (95.6) | 43,569  (95.5) | 173,198  (95.7) |  | 163,357  (95.3) | 53,410  (96.5) |  |
| **At Least One STI during Pregnancy, n (%)** | 1.78 |  |  |  |  |  |  |  |
| Yes |  | 49,291 (21.7) | 12,536  (27.5) | 36,755  (20.3) | **<0.0001** | 36,626  (21.4) | 12,665  (22.9) | **<0.0001** |
| No |  | 173,353 (76.5) | 32,717  (71.7) | 140,636  (77.7) |  | 131,742  (76.9) | 41,611  (75.2) |  |
| Percent missing is denoted as the percentage of observation that are multiply imputed (10 imputed data sets)  Bolded *P-values* denote statistical significance at α = 0.05. SD=standard deviation | | | | | | | | |

**Table S2. Linear Regression Coefficients for Regression of Birthweight, Gestational Age, and Size-for-Gestational-Age on Exposure to the Flint Water Crisis Emergency Declaration in Michigan (n=226,672)**

|  | **Birthweight** | | | **Gestational Age** | | | **Size-for-Gestational-Age** | | |  |
| --- | --- | --- | --- | --- | --- | --- | --- | --- | --- | --- |
|  | **Model 5^b^** | | | **Model 5^b^** | | | **Model 5^b^** | | |  |
| **Variable** | **Beta** | **SE** | ***P-value*** | **Beta** | **SE** | ***P-value*** | **Beta** | **SE** | ***P-value*** |  |
| **Intercept** | 3204.38 | 9.31 | **<0.0001** | 39.57 | 0.03 | **<0.0001** | -0.38 | 0.02 | **<0.0001** |  |
| **Exposed^a^** |  |  |  |  |  |  |  |  |  |  |
| Yes | -17.89 | 2.96 | **<0.0001** | 0.01 | 0.01 | 0.5220 | -0.04 | 0.01 | **<0.0001** |  |
| No | Ref |  |  | Ref |  |  | Ref |  |  |  |
| **Birthing Parent Race** |  |  |  |  |  |  |  |  |  |  |
| Non-Hispanic Black | -238.81 | 3.77 | **<0.0001** | -0.44 | 0.01 | **<0.0001** | -0.38 | 0.01 | **<0.0001** |  |
| Non-Hispanic White | Ref |  |  | Ref |  |  | Ref |  |  |  |
| **Birthing Parent Age** | -3.03 | 0.27 | **<0.0001** | -0.02 | 0.001 | **<0.0001** | -0.001 | 0.001 | 0.0887 |  |
| **Birthing Parent Education** |  |  |  |  |  |  |  |  |  |  |
| < High School | -115.03 | 4.88 | **<0.0001** | -0.24 | 0.02 | **<0.0001** | -0.17 | 0.01 | **<0.0001** |  |
| High School/GED | -71.38 | 3.59 | **<0.0001** | -0.18 | 0.01 | **<0.0001** | -0.10 | 0.01 | **<0.0001** |  |
| Some College | -30.90 | 3.25 | **<0.0001** | -0.09 | 0.01 | **<0.0001** | -0.04 | 0.01 | **<0.0001** |  |
| College or More | Ref |  |  | Ref |  |  | Ref |  |  |  |
| **Birthing Parent Marital Status** |  |  |  |  |  |  |  |  |  |  |
| Never Married | -43.04 | 3.13 | **<0.0001** | -0.12 | 0.01 | **<0.0001** | -0.04 | 0.01 | **<0.0001** |  |
| Married | Ref |  |  | Ref |  |  | Ref |  |  |  |
| Divorced/Widowed | -67.25 | 6.81 | **<0.0001** | -0.21 | 0.02 | **<0.0001** | -0.07 | 0.01 | **<0.0001** |  |
| **Source of Payment for Delivery** |  |  |  |  |  |  |  |  |  |  |
| Private Insurance | Ref |  |  | Ref |  |  | Ref |  |  |  |
| Medicaid | -43.98 | 3.08 | **<0.0001** | -0.08 | 0.01 | **<0.0001** | -0.07 | 0.01 | **<0.0001** |  |
| Self-Pay | 48.56 | 9.72 | **<0.0001** | 0.22 | 0.04 | **<0.0001** | 0.08 | 0.02 | **<0.0001** |  |
| Other | -33.85 | 12.10 | **0.0052** | -0.05 | 0.04 | 0.2238 | -0.05 | 0.02 | **0.0166** |  |
| **Receipt of WIC During Pregnancy** |  |  |  |  |  |  |  |  |  |  |
| Yes | 2.57 | 30.9 | 0.4054 | 0.10 | 0.01 | **<0.0001** | -0.05 | 0.01 | **<0.0001** |  |
| No | Ref |  |  | Ref |  |  | Ref |  |  |  |
| **Pre-pregnancy BMI** | 10.32 | 0.18 | **<0.0001** | 0.01 | 0.001 | **<0.0001** | 0.02 | 0.0003 | **<0.0001** |  |
| **Parity (including birth on record)** | 18.41 | 0.78 | **<0.0001** | -0.04 | 0.003 | **<0.0001** | 0.05 | 0.001 | **<0.0001** |  |
| **Infant's Sex** |  |  |  |  |  |  |  |  |  |  |
| Female | 121.59 | 2.27 | **<0.0001** | -0.09 | 0.01 | **<0.0001** | 0.02 | 0.004 | **<0.0001** |  |
| Male | Ref |  |  | Ref |  |  | Ref |  |  |  |
| **Residential Geographic Region** |  |  |  |  |  |  |  |  |  |  |
| Upper Peninsula Prosperity Alliance | 48.83 | 7.49 | **<0.0001** | 0.13 | 0.03 | **<0.0001** | 0.08 | 0.01 | **<0.0001** |  |
| Northwest Prosperity Region | 52.78 | 7.11 | **<0.0001** | 0.18 | 0.03 | **<0.0001** | 0.08 | 0.01 | **<0.0001** |  |
| Northeast Prosperity Region | 31.77 | 9.06 | **0.0005** | 0.10 | 0.03 | **0.0032** | 0.05 | 0.02 | **0.0028** |  |
| West Michigan Prosperity Alliance | 33.85 | 3.41 | **<0.0001** | 0.03 | 0.01 | **0.0046** | 0.07 | 0.01 | **<0.0001** |  |
| East Central Michigan Prosperity Region | 24.13 | 5.30 | **<0.0001** | 0.11 | 0.02 | **<0.0001** | 0.02 | 0.01 | **0.0291** |  |
| East Michigan Prosperity Region | 5.52 | 4.60 | 0.2298 | -0.08 | 0.02 | **<0.0001** | 0.04 | 0.01 | **<0.0001** |  |
| South Central Prosperity Region | 26.57 | 5.64 | **<0.0001** | 0.09 | 0.02 | **<0.0001** | 0.04 | 0.01 | **0.0001** |  |
| Southwest Prosperity Region | 32.26 | 4.41 | **<0.0001** | 0.20 | 0.02 | **<0.0001** | 0.02 | 0.01 | **0.0503** |  |
| Southeast Michigan Prosperity Region | 20.33 | 4.17 | **<0.0001** | 0.08 | 0.01 | **<0.0001** | 0.02 | 0.01 | **0.0055** |  |
| Detroit Metro Prosperity Region | Ref |  |  | Ref |  |  | Ref |  |  |  |
| **Kessner Index** |  |  |  |  |  |  |  |  |  |  |
| Adequate | Ref |  |  | Ref |  |  | Ref |  |  |  |
| Intermediate | -44.68 | 2.83 | **<0.0001** | -0.06 | 0.01 | **<0.0001** | -0.07 | 0.01 | **<0.0001** |  |
| Inadequate | -107.56 | 4.24 | **<0.0001** | -0.33 | 0.02 | **<0.0001** | -0.09 | 0.01 | **<0.0001** |  |
| **Tobacco Use during Pregnancy** |  |  |  |  |  |  |  |  |  |  |
| Yes | -111.53 | 3.03 | **<0.0001** | -0.08 | 0.01 | **<0.0001** | -0.23 | 0.01 | **<0.0001** |  |
| No | Ref |  |  | Ref |  |  | Ref |  |  |  |
| **Alcohol Use during Pregnancy** |  |  |  |  |  |  |  |  |  |  |
| Yes | -71.56 | 13.57 | **<0.0001** | -0.12 | 0.05 | 0.0109 | -0.09 | 0.02 | **0.0005** |  |
| No | Ref |  |  | Ref |  |  | Ref |  |  |  |
| **Gestational Diabetes** |  |  |  |  |  |  |  |  |  |  |
| Yes | 13.92 | 5.38 | **0.0097** | -0.35 | 0.02 | **<0.0001** | 0.12 | 0.01 | **<0.0001** |  |
| No | Ref |  |  | Ref |  |  | Ref |  |  |  |
| **Pre-Pregnancy Diabetes, Pre-Pregnancy Hypertension and/or Gestational Hypertension** |  |  |  |  |  |  |  |  |  |  |
| Yes | -244.83 | 4.33 | **<0.0001** | -1.11 | 0.02 | **<0.0001** | -0.15 | 0.01 | **<0.0001** |  |
| No | Ref |  |  | Ref |  |  | Ref |  |  |  |
| **Previous Preterm Birth and/or Other Previous Poor Pregnancy Outcome** |  |  |  |  |  |  |  |  |  |  |
| Yes | -230.02 | 5.83 | **<0.0001** | -0.97 | 0.02 | **<0.0001** | -0.13 | 0.01 | **<0.0001** |  |
| No | Ref |  |  | Ref |  |  | Ref |  |  |  |
| **At Least One STI during Pregnancy** |  |  |  |  |  |  |  |  |  |  |
| Yes | 24.35 | 2.80 | **<0.0001** | 0.13 | 0.01 | **<0.0001** | 0.0003 | 0.01 | 0.9473 |  |
| No | Ref |  |  | Ref |  |  |  |  |  |  |
| **Interaction** |  |  |  |  |  |  |  |  |  |  |
| Race*Exposure | -12.44 | 6.61 | 0.0599 | -0.06 | 0.02 | **0.0073** | -0.01 | 0.01 | 0.6366 |  |
| ^a^ Exposed infants were born between 1/5/2016 and 9/30/2016; unexposed infants were born in the same 37-week period in 2013, 2014, or 2015.  ^b^ Linear regression model adjusted for covariates, interaction, and the following hypothesized mediators: Kessner index, tobacco use during pregnancy, alcohol use during pregnancy, gestational diabetes, pre-pregnancy diabetes/pre-pregnancy hypertension/gestational hypertension, previous preterm birth or other poor pregnancy outcome, and at least one STI during pregnancy.  Bolded *P-values* denote statistical significance at α = 0.05. | | | | | | | | | | |

| **Table S3. Predicted Percentages and Adjusted Odds Ratios (aOR) with 95% Confidence Intervals (CI) for Low Birthweight, Preterm Birth, and Small-for-Gestational-Age from Logistic Regression of Birth Outcomes on Exposure to the Flint Water Crisis Emergency Declaration in Michigan (n=226,672)** | | | | | | | |  |
| --- | --- | --- | --- | --- | --- | --- | --- | --- |
|  | Unexposed^a^ n=171,328 | | Exposed^a^ n=55,344 | | Race x Exposure^c^ | Race | Exposure | |
|  | Non-Hispanic Black n=34,540 | Non-Hispanic White n=136,788 | Non-Hispanic Black n=11,073 | Non-Hispanic White n=44,271 | *P-value* | *P-value* | *P-value* | |
| **Low Birthweight, % (95% CI)** | | | | | | | |  |
| Model 3^b^ | 11.2 (10.4, 11.9) | 5.9 (5.5, 6.2) | 12.0 (11.1, 13.0) | 6.2 (5.7, 6.6) | 0.4677 | **<0.0001** | **0.0397** | |
| NHB vs. NHW, aOR (95% CI) | 2.02 (1.92, 2.12) | | 2.08 (1.93, 2.24) | |  |  |  | |
| **Preterm Birth, % (95% CI)** | | | | | | | |  |
| Model 3^b^ | 11.6 (10.9, 12.3) | 7.6 (7.2, 8.1) | 12.0 (11.1, 12.9) | 7.8 (7.4, 8.3) | 0.7012 | **<0.0001** | 0.2187 | |
| NHB vs. NHW, aOR (95% CI) | 1.59 (1.52, 1.66) | | 1.61 (1.50, 1.73) | |  |  |  | |
| **Small for Gestational Age, % (95% CI)** | | | | | | | |  |
| Model 3^b^ | 15.4 (14.6, 16.3) | 9.1 (8.7, 9.6) | 17.0 (15.9, 18.0) | 9.4 (8.9, 10.0) | **0.0416** | **<0.0001** | **0.0387** | |
| NHB vs. NHW, aOR (95% CI) | 1.82 (1.75, 1.89) | | 1.96 (1.83, 2.08) | |  |  |  | |
| ^a^ Exposed infants were born between 1/5/2016 and 9/30/2016; unexposed infants were born in the same 37-week period in 2013, 2014, or 2015.  ^b^ Logistic regression model adjusted for covariates only: birthing parent age, education, marital status, source of payment for delivery, receipt of WIC during pregnancy, pre-pregnancy BMI, parity, infant sex, and residential geographic region.  ^c^ Interaction term *P-value* comes from Model 4  Bolded *P-values* denote statistical significance at α = 0.05. | | | | | | | |  |

| **Table S4. Predicted Means and 95% Confidence Intervals (CI) for Birthweight, Gestational Age, and Size-for-Gestational-Age, 2015 vs. 2016, from Linear Regression of Birth Outcomes on Exposure to the Flint Water Crisis Emergency Declaration in Michigan (n=112,998)** | | | | | | | |
| --- | --- | --- | --- | --- | --- | --- | --- |
|  | Unexposed: 2015^a^ n=57,654 | | Exposed: 2016^a^ n=55,344 | | Race x Exposure^c^ | Race^b^ | Exposure^b^ |
|  | Non-Hispanic Black n=11,500 | Non-Hispanic White n=46,154 | Non-Hispanic Black n=11,073 | Non-Hispanic White n=44,271 | *P-value* | *P-value* | *P-value* |
| **Birthweight, grams (95% CI)** | | | | | | | |
| Model 3^b^ | 3,121.4  (3,052.0, 3,190.9) | 3,333.7  (3,317.3, 3,350.0) | 3,098.8  (3,029.3, 3,168.3) | 3,321.6  (3,305.2, 3,337.9) | 0.1370 | **<0.0001** | **0.0007** |
| **Gestational Age, weeks (95% CI)** | | | | | | | |
| Model 3^b^ | 38.41  (38.13, 38.70) | 38.84  (38.78, 38.90) | 38.38  (38.10, 38.67) | 38.83  (38.77, 38.88) | 0.4632 | **<0.0001** | 0.2183 |
| **Size for Gestational Age z-score, mean (95% CI)** | | | | | | | |
| Model 3^b^ | -0.28  (-0.39, -0.16) | 0.04  (0.01, 0.07) | -0.31  (-0.42, -0.20) | 0.02  (-0.01, 0.05) | 0.3022 | **<0.0001** | **<0.0001** |
| ^a^ Exposed infants were born between 1/5/2016 and 9/30/2016; unexposed infants were born in the same 37-week period in 2015.  ^b^ Linear regression model adjusted for covariates only: birthing parent age, education, marital status, source of payment for delivery, receipt of WIC during pregnancy, pre-pregnancy BMI, parity, infant sex, and residential geographic region.  ^c^ Interaction term *P-value* comes from Model 4  Bolded *P-values* denote statistical significance at α = 0.05. | | | | | | | |

| **Table S5. Predicted Means and 95% Confidence Intervals for Birthweight, Gestational Age, and Size-for-Gestational-Age, 2014 vs. 2016, from Linear Regression of Birth Outcomes on Exposure to the Flint Water Crisis Emergency Declaration in Michigan (n=113,944)** | | | | | | | |
| --- | --- | --- | --- | --- | --- | --- | --- |
|  | Unexposed: 2014^a^ n=58,600 | | Exposed: 2016^a^ n=55,344 | | Race x Exposure^c^ | Race^b^ | Exposure^b^ |
|  | Non-Hispanic Black n=11,596 | Non-Hispanic White n=47,004 | Non-Hispanic Black n=11,073 | Non-Hispanic White n=44,271 | *P-value* | *P-value* | *P-value* |
| **Birthweight, grams (95% CI)** | | | | | | | |
| Model 3^b^ | 3,237.4  (3,224.6, 3250.2) | 3,336.1  (3,320.8, 3351.4) | 3,218.9  (3205.8, 3231.9) | 3,319.5  (3,319.5, 3,334.9) | 0.5857 | **<0.0001** | **<0.0001** |
| **Gestational Age, weeks (95% CI)** | | | | | | | |
| Model 3^b^ | 38.36  (38.11, 38.61) | 38.85  (38.79, 38.90) | 38.32  (38.07, 38.57) | 38.85  (38.80, 38.90) | **0.0439** | **<0.0001** | 0.6961 |
| **Size for Gestational Age z-score, mean (95% CI)** | | | | | | | |
| Model 3^b^ | -0.28  (-0.38, -0.18) | 0.04  (0.01, 0.07) | -0.31  (-0.41, -0.21) | 0.01  (-0.02, 0.03) | 0.1403 | **<0.0001** | **<0.0001** |
| ^a^ Exposed infants were born between 1/5/2016 and 9/30/2016; unexposed infants were born in the same 37-week period in 2014.  ^b^ Linear regression model adjusted for covariates only: birthing parent age, education, marital status, source of payment for delivery, receipt of WIC during pregnancy, pre-pregnancy BMI, parity, infant sex, and residential geographic region.  ^c^ Interaction term *P-value* comes from Model 4  Bolded *P-values* denote statistical significance at α = 0.05. | | | | | | | |

| **Table S6. Predicted Means and 95% Confidence Intervals for Birthweight, Gestational Age, and Size-for-Gestational-Age, 2013 vs. 2016, from Linear Regression of Birth Outcomes on Exposure to the Flint Water Crisis Emergency Declaration in Michigan (n=110,418)** | | | | | | | |
| --- | --- | --- | --- | --- | --- | --- | --- |
|  | Unexposed: 2013^a^ n=55,074 | | Exposed: 2016^a^ n=55,344 | | Race x Exposure^c^ | Race^b^ | Exposure^b^ |
|  | Non-Hispanic Black n=11,444 | Non-Hispanic White n=43,630 | Non-Hispanic Black n=11,073 | Non-Hispanic White n=44,271 | *P-value* | *P-value* | *P-value* |
| **Birthweight, grams (95% CI)** | | | | | | | |
| Model 3^b^ | 3,120.2  (3,204.8, 3,235.8) | 3,356.4  (3,340.0, 3,372.8) | 3,092.4  (3,021.0, 3,163.7) | 3,334.0  (3,317.7, 3,350.4) | 0.2961 | **<0.0001** | **<0.0001** |
| **Gestational Age, weeks (95% CI)** | | | | | | | |
| Model 3^b^ | 38.48  (38.18, 38.77) | 38.83  (38.78, 38.89) | 38.42  (38.13, 38.71) | 38.85  (38.79, 38.90) | **0.0050** | **<0.0001** | 0.9380 |
| **Size for Gestational Age z-score, mean (95% CI)** | | | | | | | |
| Model 3^b^ | -0.28  (-0.40, -0.17) | 0.09  (0.06, 0.12) | -0.33  (-0.44, -0.21) | 0.04  (0.01, 0.07) | 0.6568 | **<0.0001** | **<0.0001** |
| ^a^ Exposed infants were born between 1/5/2016 and 9/30/2016; unexposed infants were born in the same 37-week period in 2013.  ^b^ Linear regression model adjusted for covariates only: birthing parent age, education, marital status, source of payment for delivery, receipt of WIC during pregnancy, pre-pregnancy BMI, parity, infant sex, and residential geographic region.  ^c^ Interaction term *P-value* comes from Model 4  Bolded *P-values* denote statistical significance at α = 0.05. | | | | | | | |

| **Table S7. Predicted Means and 95% Confidence Intervals for Birthweight, Gestational Age, and Size-for-Gestational-Age, 2013, 2014, and 2015 vs. 2016, from Linear Regression of Birth Outcomes on Exposure to the Flint Water Crisis Emergency Declaration in Michigan (n=226,672)** | | | | | | | | | | | | | | | | |
| --- | --- | --- | --- | --- | --- | --- | --- | --- | --- | --- | --- | --- | --- | --- | --- | --- |
|  | Unexposed: 2013^a^ n=55,074 | | Unexposed: 2014^a^ n=58,600 | | Unexposed: 2015^a^ n=57,654 | | | Exposed: 2016^a^ n=55,344 | | | Race x Year^c^ | | Race^b^ | | Year^b^ | |
|  | Non-Hispanic Black n=11,444 | Non-Hispanic  White n=43,630 | Non-Hispanic Black n=11,596 | Non-Hispanic  White n=47,004 | Non-Hispanic  Black n=11,500 | Non-Hispanic  White n=44,271 | Non-Hispanic Black n=11,073 | | Non-Hispanic  White n=44,271 | *P-value* | | *P-value* | | *P-value* | |  |
| **Birthweight, grams (95% CI)** | | | | | | | | | | | | | | | | |
| Model 3^b^ | 3,133.4  (3,086.2, 3,180.7) | 3,348.6  (3,336.9, 3,360.4) | 3,130.5  (3,083.9, 3,177.2) | 3,342.6  (3,331.0, 3,354.2) | 3,127.7  (3,080.5, 3,175.0) | 3,338.5  (3,326.9, 3,350.2) | 3,105.3  (3,058.0, 3,152.6) | | 3,326.2  (3,314.5, 3,337.9) | 0.3007 | | **<0.0001** | | **<0.0001** | |  |
| **Gestational Age, weeks (95% CI)** | | | | | | | | | | | | | | | | |
| Model 3^b^ | 38.41  (38.11, 38.61) | 38.84  (38.80, 38.88) | 38.39  (38.20, 38.59) | 38.85  (38.81, 38.89) | 38.38  (38.19, 38.57) | 38.87  (38.83, 38.91) | 38.35  (38.16, 38.55) | | 38.85  (38.81, 38.90) | **0.0033** | | **<0.0001** | | 0.8880 | |  |
| **Size for Gestational Age z-score, mean (95% CI)** | | | | | | | | | | | | | | | | |
| Model 3^b^ | -0.25  (-0.32, -0.17) | 0.08  (0.05, 0.10) | -0.25  (-0.32, -0.17) | 0.06  (0.04, 0.08) | -0.25  (-0.33, -0.18) | 0.05  (0.02, 0.07) | -0.29  (-0.37, -0.21) | | 0.02  (0.003, 0.05) | 0.5953 | | **<0.0001** | | **<0.0001** | |  |
| ^a^ Exposed infants were born between 1/5/2016 and 9/30/2016; unexposed infants were born in the same 37-week period in 2013, 2014, or 2015.  ^b^ Linear regression model adjusted for covariates only: birthing parent age, education, marital status, source of payment for delivery, receipt of WIC during pregnancy, pre-pregnancy BMI, parity, infant sex, and residential geographic region.  ^c^ Interaction term *P-value* comes from Model 4  Bolded *P-values* denote statistical significance at α = 0.05. | | | | | | | | | | | | | | | | |

| **Table S8. Predicted Means and 95% Confidence Intervals (CI) for Birthweight, Gestational Age, and Size-for-Gestational-Age from Linear Regression of Birth Outcomes on Exposure to the Flint Water Crisis Emergency Declaration during the First Trimester of Pregnancy in Michigan (n=86,618)** | | | | | | | |
| --- | --- | --- | --- | --- | --- | --- | --- |
|  | Unexposed^a^ n=65,662 | | Exposed^a^ n=20,956 | | Race x Exposure^c^ | Race^b^ | Exposure^b^ |
|  | Non-Hispanic Black n=13,035 | Non-Hispanic White n=52,627 | Non-Hispanic Black n=4,265 | Non-Hispanic White n=16,691 | *P-value* | *P-value* | *P-value* |
| **Birthweight, grams (95% CI)** | | | | | | | |
| Model 3 | 3,129.7  (3,037.8, 3,221.7) | 3,327.9  (3,310.9, 3,344.9) | 3,104.0  (3,010.8, 3,197.2) | 3,308.7  (3,290.4, 3,327.0) | 0.3417 | **<0.0001** | **0.0001** |
| **Gestational Age, weeks (95% CI)** | | | | | | | |
| Model 3^b^ | 38.37  (38.00, 38.75) | 38.79  (38.73, 39.84) | 38.34  (37.97, 38.72) | 38.78  (38.71, 38.84) | 0.3923 | **<0.0001** | 0.4181 |
| **Size for Gestational Age z-score, mean (95% CI)** | | | | | | | |
| Model 3^b^ | -0.22  (-0.37, -0.07) | 0.04  (0.01, 0.08) | -0.28  (-0.43, -0.12) | 0.01  (-0.03, 0.04) | 0.3277 | **<0.0001** | **<0.0001** |
| ^a^ Exposed infants were conceived between 9/30/2015 through 12/31/2015; unexposed infants were conceived in the same period in 2012, 2013, or 2014; as well as exposed infants were conceived between 1/1/2016 and 1/5/2016, and unexposed infants were conceived during the same period in 2013, 2014, or 2015.  ^b^ Linear regression model adjusted for covariates only: birthing parent age, education, marital status, source of payment for delivery, receipt of WIC during pregnancy, pre-pregnancy BMI, parity, infant sex, and residential geographic region.  ^c^ Interaction term P-value comes from Model 4  Bolded *P-values* denote statistical significance at α = 0.05. | | | | | | | |

| **Table S9. Predicted Means and 95% Confidence Intervals (CI) for Birthweight, Gestational Age, and Size-for-Gestational-Age from Linear Regression of Birth Outcomes on Exposure to the Flint Water Crisis Emergency Declaration during the Second Trimester of Pregnancy in Michigan (n=84,505)** | | | | | | | |
| --- | --- | --- | --- | --- | --- | --- | --- |
|  | Unexposed^a^ n=63,880 | | Exposed^a^ n=20,625 | | Race x Exposure | Race | Exposure |
|  | Non-Hispanic Black n=12,495 | Non-Hispanic White n=51,385 | Non-Hispanic Black n=3,950 | Non-Hispanic White n=16,675 | *P-value* | *P-value* | *P-value* |
| **Birthweight, grams (95% CI)** | | | | | | | |
| Model 3^b^ | 3,189.0  (3,114.1, 3,263.8) | 3,365.7  (3,347.5, 3,384.0) | 3,153.2  (3,076.7, 3,229.7) | 3,352.7  (3,333.3, 3,372.1) | **0.0280** | **<0.0001** | **<0.0001** |
| **Gestational Age, weeks (95% CI)** | | | | | | | |
| Model 3^b^ | 38.74  (38.45, 39.03) | 38.91  (38.86, 38.98) | 38.71  (38.41, 39.00) | 39.94  (38.88, 39.01) | 0.1436 | **<0.0001** | 0.3927 |
| **Size for Gestational Age z-score, mean (95% CI)** | | | | | | | |
| Model 3^b^ | -0.25  (-0.38, -0.12) | 0.08  (0.05, 0.12) | -0.31  (-0.44, -0.17) | 0.05  (0.01, 0.09) | 0.2030 | **<0.0001** | **<0.0001** |
| ^a^ Exposed infants were conceived between 6/24/2015 and 9/29/2015; unexposed infants were conceived in the same period in 2012, 2013, or 2014.  ^b^ Linear regression model adjusted for covariates only: birthing parent age, education, marital status, source of payment for delivery, receipt of WIC during pregnancy, pre-pregnancy BMI, parity, infant sex, and residential geographic region.  ^c^ Interaction term P-value comes from Model 4  Bolded *P-*values denote statistical significance at α = 0.05. | | | | | | | |

| **Table S10. Predicted Means and 95% Confidence Intervals (CI) for Birthweight, Gestational Age, and Size-for-Gestational-Age from Linear Regression of Birth Outcomes on Exposure to the Flint Water Crisis Emergency Declaration during the Third Trimester of Pregnancy in Michigan (n=59,329)** | | | | | | | |
| --- | --- | --- | --- | --- | --- | --- | --- |
|  | Unexposed^a^ n=38,704 | | Exposed^a^ n=20,625 | Race x Exposure^c^ | Race^b^ | | Exposure^b^ |
|  | Non-Hispanic Black n=8,122 | Non-Hispanic White n=30,582 | Non-Hispanic Black n=2,571 | Non-Hispanic White n=10,132 | *P-value* | *P-value* | *P-value* |
| **Birthweight, grams (95% CI)** | | | | | | | |
| Model 3^b^ | 3,197.3  (3,118.5, 3,276.1) | 3,398.3  (3,377.0, 3,419.7) | 3,186.6  (3105.9, 3,267.2) | 3,381.4  (3,358.7, 3,404.0) | 0.6736 | **<0.0001** | **0.0012** |
| **Gestational Age, weeks (95% CI)** | | | | | | | |
| Model 3^b^ | 38.68  (38.43, 38.93) | 39.22  (39.15, 39.28) | 38.58  (38.31, 38.83) | 39.22  (39.15, 39.29) | **0.0035** | **<0.0001** | 0.1834 |
| **Size for Gestational Age z-score, mean (95% CI)** | | | | | | | |
| Model 3^b^ | -0.25  (-0.41, -0.09) | 0.05  (0.003, 0.09) | -0.24  (-0.40, -0.07) | 0.01  (-0.03, 0.06) | 0.0528 | **<0.0001** | **0.0135** |
| ^a^ Exposed infants were conceived between 3/20/2015 and 6/23/2015; unexposed infants were conceived in the same period in 2012, 2013, or 2014.  ^b^ Linear regression model adjusted for covariates only: birthing parent age, education, marital status, source of payment for delivery, receipt of WIC during pregnancy, pre-pregnancy BMI, parity, infant sex, and residential geographic region.  ^c^ Interaction term P-value comes from Model 4  Bolded *P-values* denote statistical significance at α = 0.05. | | | | | | | |

**Figure S1. Inclusion Flowchart**

**Figure S2. Adjusted Means for Birthweight (A), Gestational Age (B), and Size-for-Gestational-Age (C) by Maternal Race and Year in Michigan, 2013-2016 (n=226,672)**

Note: The race-by-year interactions at α = 0.05 are only statistically significant for gestational age in weeks. Means are from fully adjusted models (covariates and mediators).
