# Supplementary material for: Vicarious structural racism and infant health disparities in Michigan: The Flint Water Crisis
Source: Front Public Health. 2022 Sep 6;10:954896. doi: 10.3389/fpubh.2022.954896 (PMC9486078; doi:10.3389/fpubh.2022.954896)
Supplement: Supplementary file 2 [file Data_Sheet_2.docx]

**Table S11. Linear Regression Coefficients for Regression of Birthweight (grams) on Exposure to the Flint Water Crisis Emergency Declaration in Michigan Excluding all of Genesee County (n=220,985).**

|  | **Model 1** | | | **Model 2** | | | **Model 3** | | | **Model 4** | | | |  |
| --- | --- | --- | --- | --- | --- | --- | --- | --- | --- | --- | --- | --- | --- | --- |
| **Variable** | **Beta** | **SE** | ***P-value*** | **Beta** | **SE** | ***P-value*** | **Beta** | **SE** | ***P-value*** | **Beta** | **SE** | ***P-value*** |  |  |
| **Intercept** | 3336.18 | 1.40 | **<0.0001** | 3388.27 | 1.33 | **<0.0001** | 3248.32 | 9.39 | **<0.0001** | 3247.67 | 9.40 | **<0.0001** |  |  |
| **Exposed^a^** |  |  |  |  |  |  |  |  |  |  |  |  |  |  |
| Yes | -16.7 | 2.84 | **<0.0001** |  |  |  | -18.86 | 2.73 | **<0.0001** | -16.62 | 3.05 | **<0.0001** |  |  |
| No | Ref |  |  |  |  |  | Ref |  |  | Ref |  |  |  |  |
| **Birthing Parent Race** |  |  |  |  |  |  |  |  |  |  |  |  |  |  |
| Non-Hispanic Black |  |  |  | -277.43 | 2.97 | **<0.0001** | -225.01 | 3.45 | **<0.0001** | -222.30 | 3.83 | **<0.0001** |  |  |
| Non-Hispanic White |  |  |  | Ref |  |  | Ref |  |  | Ref |  |  |  |  |
| **Birthing Parent Age** |  |  |  |  |  |  | -4.03 | 0.28 | **<0.0001** | -4.02 | 0.28 | **<0.0001** |  |  |
| **Birthing Parent Education** |  |  |  |  |  |  |  |  |  |  |  |  |  |  |
| < High School |  |  |  |  |  |  | -154.17 | 4.94 | **<0.0001** | -154.26 | 4.94 | **<0.0001** |  |  |
| High School/GED |  |  |  |  |  |  | -94.58 | 3.64 | **<0.0001** | -94.56 | 3.64 | **<0.0001** |  |  |
| Some College |  |  |  |  |  |  | -46.59 | 3.33 | **<0.0001** | -46.59 | 3.33 | **<0.0001** |  |  |
| College or More |  |  |  |  |  |  | Ref |  |  | Ref |  |  |  |  |
| **Birthing Parent Marital Status** |  |  |  |  |  |  |  |  |  |  |  |  |  |  |
| Never Married |  |  |  |  |  |  | -65.96 | 3.18 | **<0.0001** | -65.99 | 3.18 | **<0.0001** |  |  |
| Married |  |  |  |  |  |  | Ref |  |  | Ref |  |  |  |  |
| Divorced/Widowed |  |  |  |  |  |  | -92.64 | 7.01 | **<0.0001** | -92.65 | 7.01 | **<0.0001** |  |  |
| **Source of Payment for Delivery** |  |  |  |  |  |  |  |  |  |  |  |  |  |  |
| Private Insurance |  |  |  |  |  |  | Ref |  |  | Ref |  |  |  |  |
| Medicaid |  |  |  |  |  |  | -55.81 | 3.16 | **<0.0001** | -55.65 | 3.16 | **<0.0001** |  |  |
| Self-Pay |  |  |  |  |  |  | 44.15 | 9.95 | **<0.0001** | 44.17 | 9.95 | **<0.0001** |  |  |
| Other |  |  |  |  |  |  | -30.19 | 12.33 | **0.0144** | -30.92 | 12.34 | **0.0122** |  |  |
| **Receipt of WIC During Pregnancy** |  |  |  |  |  |  |  |  |  |  |  |  |  |  |
| Yes |  |  |  |  |  |  | -0.31 | 3.18 | 0.9221 | -0.38 | 3.18 | 0.9052 |  |  |
| No |  |  |  |  |  |  | Ref |  |  | Ref |  |  |  |  |
| **Pre-pregnancy BMI** |  |  |  |  |  |  | 8.82 | 0.18 | **<0.0001** | 8.82 | 0.18 | **<0.0001** |  |  |
| **Parity (including birth on record)** |  |  |  |  |  |  | 13.87 | 0.79 | **<0.0001** | 13.87 | 0.79 | **<0.0001** |  |  |
| **Infant's Sex** |  |  |  |  |  |  |  |  |  |  |  |  |  |  |
| Female |  |  |  |  |  |  | Ref |  |  | Ref |  |  |  |  |
| Male |  |  |  |  |  |  | 121.29 | 2.34 | **<0.0001** | 121.29 | 2.34 | **<0.0001** |  |  |
| **Residential Geographic Region** |  |  |  |  |  |  |  |  |  |  |  |  |  |  |
| Upper Peninsula Prosperity Alliance |  |  |  |  |  |  | 40.84 | 7.60 | **<0.0001** | 40.86 | 7.60 | **<0.0001** |  |  |
| Northwest Prosperity Region |  |  |  |  |  |  | 54.68 | 7.22 | **<0.0001** | 54.66 | 7.22 | **<0.0001** |  |  |
| Northeast Prosperity Region |  |  |  |  |  |  | 24.40 | 9.20 | **0.0080** | 24.41 | 9.20 | **0.0080** |  |  |
| West Michigan Prosperity Alliance |  |  |  |  |  |  | 30.88 | 3.46 | **<0.0001** | 30.89 | 3.46 | **<0.0001** |  |  |
| East Central Michigan Prosperity Region |  |  |  |  |  |  | 16.09 | 5.39 | **0.0174** | 16.11 | 5.39 | **0.0028** |  |  |
| East Michigan Prosperity Region |  |  |  |  |  |  | 13.61 | 5.56 | **0.0028** | 15.04 | 5.56 | **0.0142** |  |  |
| South Central Prosperity Region |  |  |  |  |  |  | 15.06 | 5.73 | **0.0143** | 19.77 | 5.73 | **0.0086** |  |  |
| Southwest Prosperity Region |  |  |  |  |  |  | 19.77 | 4.45 | **<0.0001** | 16.35 | 4.45 | **<0.0001** |  |  |
| Southeast Michigan Prosperity Region |  |  |  |  |  |  | 16.33 | 4.24 | **0.0001** | 13.64 | 4.24 | **0.0001** |  |  |
| Detroit Metro Prosperity Region |  |  |  |  |  |  | Ref |  |  | Ref |  |  |  |  |
| **Interaction** |  |  |  |  |  |  |  |  |  |  |  |  |  |  |
| Race*Exposure |  |  |  |  |  |  |  |  |  | -11.13 | 6.80 | 0.1019 |  |  |
| ^a^ Exposed infants were born between 1/5/2016 and 9/30/2016; unexposed infants were born in the same 37-week period in 2013, 2014, or 2015.  Bolded *P-values* denote statistical significance at α = 0.05.  Sample Sizes Non-Hispanic Black: 33,930 (unexposed), 10,789 (exposed); Non-Hispanic White: 133,418 (unexposed), 42,839 (exposed). | | | | | | | | | | | | | | |

**Table S12 Linear Regression Coefficients for Regression of Gestational Age (weeks) on Exposure to the Flint Water Crisis Emergency Declaration in Michigan Excluding all of Genesee County (n=220,985).**

|  | **Model 1** | | | **Model 2** | | | **Model 3** | | | **Model 4** | | | |  |
| --- | --- | --- | --- | --- | --- | --- | --- | --- | --- | --- | --- | --- | --- | --- |
| **Variable** | **Beta** | **SE** | ***P-value*** | **Beta** | **SE** | ***P-value*** | **Beta** | **SE** | ***P-value*** | **Beta** | **SE** | ***P-value*** |  |  |
| **Intercept** | 38.77 | 0.005 | **<0.0001** | 38.87 | 0.005 | **<0.0001** | 39.84 | 0.03 | **<0.0001** | 39.84 | 0.03 | **<0.0001** |  |  |
| **Exposed^a^** |  |  |  |  |  |  |  |  |  |  |  |  |  |  |
| Yes | -0.009 | 0.01 | 0.3523 |  |  |  | -0.01 | 0.01 | 0.3440 | 0.002 | 0.01 | 0.8530 |  |  |
| No | Ref |  |  |  |  |  | Ref |  |  | Ref |  |  |  |  |
| **Birthing Parent Race** |  |  |  |  |  |  |  |  |  |  |  |  |  |  |
| Non-Hispanic Black |  |  |  | -0.55 | 0.01 | **<0.0001** | -0.43 | 0.01 | **<0.0001** | -0.42 | 0.01 | **<0.0001** |  |  |
| Non-Hispanic White |  |  |  | Ref |  |  | Ref |  |  | Ref |  |  |  |  |
| **Birthing Parent Age** |  |  |  |  |  |  | -0.02 | 0.01 | **<0.0001** | -0.02 | 0.001 | **<0.0001** |  |  |
| **Birthing Parent Education** |  |  |  |  |  |  |  |  |  |  |  |  |  |  |
| < High School |  |  |  |  |  |  | -0.30 | 0.02 | **<0.0001** | -0.30 | 0.02 | **<0.0001** |  |  |
| High School/GED |  |  |  |  |  |  | -0.21 | 0.01 | **<0.0001** | -0.21 | 0.01 | **<0.0001** |  |  |
| Some College |  |  |  |  |  |  | -0.12 | 0.01 | **<0.0001** | -0.12 | 0.01 | **<0.0001** |  |  |
| College or More |  |  |  |  |  |  | Ref |  |  | Ref |  |  |  |  |
| **Birthing Parent Marital Status** |  |  |  |  |  |  |  |  |  |  |  |  |  |  |
| Never Married |  |  |  |  |  |  | -0.14 | 0.01 | **<0.0001** | -0.14 | 0.01 | **<0.0001** |  |  |
| Married |  |  |  |  |  |  | Ref |  |  | Ref |  |  |  |  |
| Divorced/Widowed |  |  |  |  |  |  | -0.25 | 0.03 | **<0.0001** | -0.25 | 0.03 | **<0.0001** |  |  |
| **Source of Payment for Delivery** |  |  |  |  |  |  |  |  |  |  |  |  |  |  |
| Private Insurance |  |  |  |  |  |  | Ref |  |  | Ref |  |  |  |  |
| Medicaid |  |  |  |  |  |  | -0.09 | 0.01 | **<0.0001** | -0.09 | 0.01 | **<0.0001** |  |  |
| Self-Pay |  |  |  |  |  |  | 0.22 | 0.04 | **<0.0001** | 0.22 | 0.04 | **<0.0001** |  |  |
| Other |  |  |  |  |  |  | -0.02 | 0.04 | 0.7335 | -0.02 | 0.04 | 0.6716 |  |  |
| **Receipt of WIC During Pregnancy** |  |  |  |  |  |  |  |  |  |  |  |  |  |  |
| Yes |  |  |  |  |  |  | 0.12 | 0.01 | **<0.0001** | 0.12 | 0.01 | **<0.0001** |  |  |
| No |  |  |  |  |  |  | Ref |  |  | Ref |  |  |  |  |
| **Pre-pregnancy BMI** |  |  |  |  |  |  | -0.002 | 0.001 | **0.0003** | -0.002 | 0.001 | **0.0003** |  |  |
| **Parity (including birth on record)** |  |  |  |  |  |  | -0.05 | 0.003 | **<0.0001** | -0.05 | 0.003 | **<0.0001** |  |  |
| **Infant's Sex** |  |  |  |  |  |  |  |  |  |  |  |  |  |  |
| Female |  |  |  |  |  |  | Ref |  |  | Ref |  |  |  |  |
| Male |  |  |  |  |  |  | -0.09 | 0.01 | <**0.0001** | -0.09 | 0.01 | **<0.0001** |  |  |
| **Residential Geographic Region** |  |  |  |  |  |  |  |  |  |  |  |  |  |  |
| Upper Peninsula Prosperity Alliance |  |  |  |  |  |  | 0.13 | 0.03 | **<0.0001** | 0.13 | 0.03 | **<0.0001** |  |  |
| Northwest Prosperity Region |  |  |  |  |  |  | 0.22 | 0.03 | **<0.0001** | 0.22 | 0.03 | **<0.0001** |  |  |
| Northeast Prosperity Region |  |  |  |  |  |  | 0.12 | 0.03 | **0.0002** | 0.12 | 0.03 | **0.0002** |  |  |
| West Michigan Prosperity Alliance |  |  |  |  |  |  | 0.03 | 0.01 | **0.0107** | 0.03 | 0.01 | **0.0106** |  |  |
| East Central Michigan Prosperity Region |  |  |  |  |  |  | 0.11 | 0.02 | **<0.0001** | 0.11 | 0.02 | **<0.0001** |  |  |
| East Michigan Prosperity Region |  |  |  |  |  |  | -0.04 | 0.02 | 0.0648 | -0.04 | 0.02 | 0.0656 |  |  |
| South Central Prosperity Region |  |  |  |  |  |  | 0.08 | 0.02 | **0.0001** | 0.08 | 0.02 | **0.0001** |  |  |
| Southwest Prosperity Region |  |  |  |  |  |  | 0.17 | 0.02 | **<0.0001** | 0.17 | 0.02 | **<0.0001** |  |  |
| Southeast Michigan Prosperity Region |  |  |  |  |  |  | 0.09 | 0.02 | **<0.0001** | 0.09 | 0.02 | **<0.0001** |  |  |
| Detroit Metro Prosperity Region |  |  |  |  |  |  | Ref |  |  | Ref |  |  |  |  |
| **Interaction** |  |  |  |  |  |  |  |  |  |  |  |  |  |  |
| Race*Exposure |  |  |  |  |  |  |  |  |  | -0.06 | 0.02 | 0.0212 |  |  |
| ^a^ Exposed infants were born between 1/5/2016 and 9/30/2016; unexposed infants were born in the same 37-week period in 2013, 2014, or 2015.  Bolded *P-values* denote statistical significance at α = 0.05.  Sample Sizes Non-Hispanic Black: 33,930 (unexposed), 10,789 (exposed); Non-Hispanic White: 133,418 (unexposed), 42,839 (exposed). | | | | | | | | | | | | | | |

**Table S13. Linear Regression Coefficients for Regression of Size for Gestational Age (z-score) on Exposure to the Flint Water Crisis Emergency Declaration in Michigan Excluding all of Genesee County (n=220,985).**

|  | **Model 1** | | | | **Model 2** | | | **Model 3** | | | **Model 4** | | |
| --- | --- | --- | --- | --- | --- | --- | --- | --- | --- | --- | --- | --- | --- |
| **Variable** | **Beta** | **SE** | ***P-value*** | **Beta** | | **SE** | ***P-value*** | **Beta** | **SE** | ***P-value*** | **Beta** | **SE** | ***P-value*** |
| **Intercept** | 0.06 | 0.003 | **<0.0001** | 0.14 | | 0.002 | **<0.0001** | -0.37 | 0.02 | **<0.0001** | -0.37 | 0.02 | **<0.0001** |
| **Exposed^a^** |  |  |  |  | |  |  |  |  |  |  |  |  |
| Yes | -0.03 | 0.01 | **<0.0001** |  | |  |  | -0.04 | 0.005 | **<0.0001** | -0.03 | 0.01 | **<0.0001** |
| No | Ref |  |  |  | |  |  | Ref |  |  | Ref |  |  |
| **Birthing Parent Race** |  |  |  |  | |  |  |  |  |  |  |  |  |
| Non-Hispanic Black |  |  |  | -0.43 | | 0.005 | **<0.0001** | -0.35 | 0.01 | **<0.0001** | -0.35 | 0.01 | **<0.0001** |
| Non-Hispanic White |  |  |  |  | |  |  | Ref |  |  | Ref |  |  |
| **Birthing parent Age** |  |  |  |  | |  |  | -0.001 | 0.001 | **0.0054** | -0.001 | 0.001 | **0.0054** |
| **Maternal Education** |  |  |  |  | |  |  |  |  |  |  |  |  |
| < High School |  |  |  |  | |  |  | -0.23 | 0.01 | **<0.0001** | -0.23 | 0.01 | **<0.0001** |
| High School/GED |  |  |  |  | |  |  | -0.14 | 0.01 | **<0.0001** | -0.14 | 0.01 | **<0.0001** |
| Some College |  |  |  |  | |  |  | -0.06 | 0.01 | **<0.0001** | -0.06 | 0.01 | **<0.0001** |
| College or More |  |  |  |  | |  |  | Ref |  |  | Ref |  |  |
| **Birthing Parent Marital Status** |  |  |  |  | |  |  |  |  |  |  |  |  |
| Never Married |  |  |  |  | |  |  | -0.09 | 0.01 | **<0.0001** | -0.09 | 0.01 | **<0.0001** |
| Married |  |  |  |  | |  |  | Ref |  |  | Ref |  |  |
| Divorced/Widowed |  |  |  |  | |  |  | -0.12 | 0.01 | **<0.0001** | -0.12 | 0.01 | **<0.0001** |
| **Source of Payment for Delivery** |  |  |  |  | |  |  |  |  |  |  |  |  |
| Private Insurance |  |  |  |  | |  |  | Ref |  |  | Ref |  |  |
| Medicaid |  |  |  |  | |  |  | -0.09 | 0.01 | **<0.0001** | -0.09 | 0.01 | **<0.0001** |
| Self-Pay |  |  |  |  | |  |  | 0.07 | 0.02 | **<0.0001** | 0.07 | 0.02 | **<0.0001** |
| Other |  |  |  |  | |  |  | -0.06 | 0.02 | **0.0082** | -0.06 | 0.02 | **0.0079** |
| **Receipt of WIC During Pregnancy** |  |  |  |  | |  |  |  |  |  |  |  |  |
| Yes |  |  |  |  | |  |  | -0.06 | 0.01 | **<0.0001** | -0.06 | 0.01 | **<0.0001** |
| No |  |  |  |  | |  |  | Ref |  |  | Ref |  |  |
| **Pre-pregnancy BMI** |  |  |  |  | |  |  | 0.02 | 0.0003 | **<0.0001** | 0.02 | 0.0003 | **<0.0001** |
| **Parity (including birth on record)** |  |  |  |  | |  |  | 0.04 | 0.001 | **<0.0001** | 0.04 | 0.001 | **<0.0001** |
| **Infant's Sex** |  |  |  |  | |  |  |  |  |  |  |  |  |
| Female |  |  |  |  | |  |  | Ref |  |  |  |  |  |
| Male |  |  |  |  | |  |  | 0.02 | 0.004 | **<0.0001** | 0.02 | 0.004 | **<0.0001** |
| **Residential Geographic Region** |  |  |  |  | |  |  |  |  |  |  |  |  |
| Upper Peninsula Prosperity Alliance |  |  |  |  | |  |  | 0.06 | 0.01 | **<0.0001** | 0.06 | 0.01 | **<0.0001** |
| Northwest Prosperity Region |  |  |  |  | |  |  | 0.07 | 0.01 | **<0.0001** | 0.07 | 0.01 | **<0.0001** |
| Northeast Prosperity Region |  |  |  |  | |  |  | 0.02 | 0.02 | 0.1919 | 0.02 | 0.02 | 0.1918 |
| West Michigan Prosperity Alliance |  |  |  |  | |  |  | 0.06 | 0.01 | **<0.0001** | 0.06 | 0.01 | **<0.0001** |
| East Central Michigan Prosperity Region |  |  |  |  | |  |  | 0.004 | 0.01 | 0.6482 | 0.004 | 0.01 | 0.6477 |
| East Michigan Prosperity Region |  |  |  |  | |  |  | 0.04 | 0.01 | **<0.0001** | 0.04 | 0.01 | **<0.0001** |
| South Central Prosperity Region |  |  |  |  | |  |  | 0.02 | 0.01 | 0.0851 | 0.02 | 0.01 | 0.0853 |
| Southwest Prosperity Region |  |  |  |  | |  |  | -0.0001 | 0.01 | 0.9943 | -0.0001 | 0.01 | 0.9942 |
| Southeast Michigan Prosperity Region |  |  |  |  | |  |  | 0.01 | 0.01 | 0.1506 | 0.01 | 0.01 | 0.1503 |
| Detroit Metro Prosperity Region |  |  |  |  | |  |  | Ref |  |  | Ref |  |  |
| **Interactions** |  |  |  |  | |  |  |  |  |  |  |  |  |
| Race*Exposure |  |  |  |  | |  |  |  |  |  | -0.01 | 0.01 | 0.6838 |
| ^a^ Exposed infants were born between 1/5/2016 and 9/30/2016; unexposed infants were born in the same 37-week period in 2013, 2014, or 2015.  Bolded *P-values* denote statistical significance at α = 0.05.  Sample Sizes Non-Hispanic Black: 33,930 (unexposed), 10,789 (exposed); Non-Hispanic White: 133,418 (unexposed), 42,839 (exposed). | | | | | | | | | | | | | |

**Table S14. Predicted Means^a^ and Mean Difference for Birth Outcomes on Exposure to the Flint Water Crisis Emergency Declaration in Michigan Excluding all of Genesee County (n=220,985).**

|  | **Birthweight (Grams)** | | **Gestational Age (Weeks)** | | **Size for Gestational Age (Z-score)** | |
| --- | --- | --- | --- | --- | --- | --- |
|  | **Non-Hispanic Black** | **Non-Hispanic White** | **Non-Hispanic Black** | **Non-Hispanic White** | **Non-Hispanic Black** | **Non-Hispanic White** |
| **Exposure Status^b^** | **Mean (95% CI)** | **Mean (95% CI)** | **Mean (95% CI)** | **Mean (95% CI)** | **Mean (95% CI)** | **Mean (95% CI)** |
| Exposed | 3104.7 | 3327.6 | 38.35 | 38.85 | -0.29 | 0.03 |
|  | (3057.3, 3152.2) | (3315.8, 3339.5) | (38.21, 38.59) | (38.81, 38.89) | (-0.36, -0.21) | (0.006, 0.05) |
| Unexposed | 3130.6 | 3344.6 | 38.40 | 38.86 | -0.25 | 0.06 |
|  | (3084.1, 3176.1) | (3333.5, 3355.7) | (38.16, 38.54) | (38.82, 38.89) | (-0.32, -0.17) | (0.04, 0.08) |
| Least Square Mean Difference | -25.8 | -17.0 | -0.05 | 0.0001 | -0.03 | -0.03 |
| (95% CI) | (-38.7, -12.8) | (-22.8, -11.1) | (-0.10, 0.01) | (-0.02, 0.02) | (-0.06, -0.02) | (-0.05, -0.02) |
| *P*-value | **0.0001** | **<0.0001** | 0.0802 | 0.9926 | **0.0005** | **<0.0001** |

^a^ Linear model (3) stratified by race and exposure status adjusted for covariates: (birthing parent age, education, marital status, source of payment for delivery, receipt of WIC during pregnancy, pre-pregnancy BMI, parity, infant sex, and residential geographic region).

^b^ Exposed infants were born between 1/5/2016 and 9/30/2016; unexposed infants were born in the same 37-week period in 2013, 2014, or 2015.

**Table S15. Linear Regression Coefficients for Regression of Birthweight (grams) on Exposure to the Flint Water Crisis Emergency Declaration in Michigan: Modified Exposure period (9/1/2015-8/30/2016=exposed) (n=239,037).**

|  | **Model 1** | | | **Model 2** | | | **Model 3** | | | **Model 4** | | | |  |
| --- | --- | --- | --- | --- | --- | --- | --- | --- | --- | --- | --- | --- | --- | --- |
| **Variable** | **Beta** | **SE** | ***P-value*** | **Beta** | **SE** | ***P-value*** | **Beta** | **SE** | ***P-value*** | **Beta** | **SE** | ***P-value*** |  |  |
| **Intercept** | 3333.30 | 1.42 | **<0.0001** | 3384.91 | 1.28 | **<0.0001** | 3246.44 | 9.06 | **<0.0001** | 3244.57 | 9.08 | **<0.0001** |  |  |
| **Exposed^a^** |  |  |  |  |  |  |  |  |  |  |  |  |  |  |
| Yes | -12.64 | 2.50 | **<0.0001** |  |  |  | -13.68 | 2.40 | **<0.0001** | -11.37 | 2.69 | **<0.0001** |  |  |
| No | Ref |  |  |  |  |  | Ref |  |  | Ref |  |  |  |  |
| **Birthing Parent Race** |  |  |  |  |  |  |  |  |  |  |  |  |  |  |
| Non-Hispanic Black |  |  |  | -275.58 | 2.86 | **<0.0001** | -223.13 | 3.31 | **<0.0001** | -219.35 | 3.85 | **<0.0001** |  |  |
| Non-Hispanic White |  |  |  | Ref |  |  | Ref |  |  | Ref |  |  |  |  |
| **Birthing Parent Age** |  |  |  |  |  |  | -4.21 | 0.27 | **<0.0001** | -4.20 | 0.27 | **<0.0001** |  |  |
| **Birthing Parent Education** |  |  |  |  |  |  |  |  |  |  |  |  |  |  |
| < High School |  |  |  |  |  |  | -156.39 | 4.74 | **<0.0001** | -156.45 | 4.74 | **<0.0001** |  |  |
| High School/GED |  |  |  |  |  |  | -92.56 | 3.52 | **<0.0001** | -92.53 | 3.52 | **<0.0001** |  |  |
| Some College |  |  |  |  |  |  | -46.85 | 3.19 | **<0.0001** | -46.84 | 3.19 | **<0.0001** |  |  |
| College or More |  |  |  |  |  |  | Ref |  |  | Ref |  |  |  |  |
| **Birthing Parent Marital Status** |  |  |  |  |  |  |  |  |  |  |  |  |  |  |
| Never Married |  |  |  |  |  |  | -66.15 | 3.05 | **<0.0001** | -66.17 | 3.05 | **<0.0001** |  |  |
| Married |  |  |  |  |  |  | Ref |  |  | Ref |  |  |  |  |
| Divorced/Widowed |  |  |  |  |  |  | -94.95 | 6.74 | **<0.0001** | -94.97 | 6.74 | **<0.0001** |  |  |
| **Source of Payment for Delivery** |  |  |  |  |  |  |  |  |  |  |  |  |  |  |
| Private Insurance |  |  |  |  |  |  | Ref |  |  | Ref |  |  |  |  |
| Medicaid |  |  |  |  |  |  | -58.11 | 3.04 | **<0.0001** | -57.99 | 3.04 | **<0.0001** |  |  |
| Self-Pay |  |  |  |  |  |  | 47.16 | 9.39 | **<0.0001** | 47.17 | 9.39 | **<0.0001** |  |  |
| Other |  |  |  |  |  |  | -30.59 | 10.66 | **0.0041** | -31.99 | 10.69 | **0.0028** |  |  |
| **Receipt of WIC During Pregnancy** |  |  |  |  |  |  |  |  |  |  |  |  |  |  |
| Yes |  |  |  |  |  |  | -1.09 | 3.05 | 0.7213 | -1.14 | 3.05 | 0.7091 |  |  |
| No |  |  |  |  |  |  | Ref |  |  | Ref |  |  |  |  |
| **Pre-pregnancy BMI** |  |  |  |  |  |  | 8.90 | 0.17 | **<0.0001** | 8.90 | 0.17 | **<0.0001** |  |  |
| **Parity (including birth on record)** |  |  |  |  |  |  | 14.34 | 0.76 | **<0.0001** | 14.34 | 0.76 | **<0.0001** |  |  |
| **Infant's Sex** |  |  |  |  |  |  |  |  |  |  |  |  |  |  |
| Female |  |  |  |  |  |  | Ref |  |  | Ref |  |  |  |  |
| Male |  |  |  |  |  |  | 122.83 | 2.25 | **<0.0001** | 122.83 | 2.25 | **<0.0001** |  |  |
| **Residential Geographic Region** |  |  |  |  |  |  |  |  |  |  |  |  |  |  |
| Upper Peninsula Prosperity Alliance |  |  |  |  |  |  | 36.71 | 7.41 | **<0.0001** | 36.73 | 7.41 | **<0.0001** |  |  |
| Northwest Prosperity Region |  |  |  |  |  |  | 56.17 | 6.99 | **<0.0001** | 56.16 | 6.99 | **<0.0001** |  |  |
| Northeast Prosperity Region |  |  |  |  |  |  | 20.78 | 8.84 | **0.0187** | 20.80 | 8.84 | **0.0186** |  |  |
| West Michigan Prosperity Alliance |  |  |  |  |  |  | 31.89 | 3.37 | **<0.0001** | 32.88 | 3.37 | **<0.0001** |  |  |
| East Central Michigan Prosperity Region |  |  |  |  |  |  | 11.43 | 5.23 | **0.0287** | 11.44 | 5.23 | **0.0285** |  |  |
| East Michigan Prosperity Region |  |  |  |  |  |  | 4.93 | 4.56 | 0.2798 | 4.92 | 4.56 | 0.2800 |  |  |
| South Central Prosperity Region |  |  |  |  |  |  | 21.78 | 5.59 | **<0.0001** | 21.76 | 5.59 | **<0.0001** |  |  |
| Southwest Prosperity Region |  |  |  |  |  |  | 30.08 | 4.33 | **<0.0001** | 30.07 | 4.33 | **<0.0001** |  |  |
| Southeast Michigan Prosperity Region |  |  |  |  |  |  | 14.37 | 4.15 | **0.0005** | 14.37 | 4.15 | **0.0005** |  |  |
| Detroit Metro Prosperity Region |  |  |  |  |  |  | Ref |  |  | Ref |  |  |  |  |
| **Interaction** |  |  |  |  |  |  |  |  |  |  |  |  |  |  |
| Race*Exposure |  |  |  |  |  |  |  |  |  | -11.53 | 5.99 | 0.0543 |  |  |
| ^a^ Exposed infants were born between 9/1/2015 and 8/31/2016; unexposed infants were born in the same 37-week period in 2013, 2014, or 2015 (9/1/2013-8/31/2014 & 9/1/2014-/8/31/2015).  Bolded *P-values* denote statistical significance at α = 0.05.  Sample Sizes Non-Hispanic Black: 32,643 (unexposed), 15,687 (exposed); Non-Hispanic White: 128,639 (unexposed), 62,068 (exposed). | | | | | | | | | | | | | | |
|  | | | | | | | | | | | | | | |

**Table S16. Linear Regression Coefficients for Regression of Gestational Age (weeks) on Exposure to the Flint Water Crisis Emergency Declaration in Michigan: Alternate Exposure period (9/1/2015-8/30/2016=exposed) (n=239,037).**

|  | **Model 1** | | | **Model 2** | | | **Model 3** | | | **Model 4** | | | |  |
| --- | --- | --- | --- | --- | --- | --- | --- | --- | --- | --- | --- | --- | --- | --- |
| **Variable** | **Beta** | **SE** | ***P-value*** | **Beta** | **SE** | ***P-value*** | **Beta** | **SE** | ***P-value*** | **Beta** | **SE** | ***P-value*** |  |  |
| **Intercept** | 38.76 | 0.004 | **<0.0001** | 38.87 | 0.01 | **<0.0001** | 39.83 | 0.03 | **<0.0001** | 39.83 | 0.03 | **<0.0001** |  |  |
| **Exposed^a^** |  |  |  |  |  |  |  |  |  |  |  |  |  |  |
| Yes | -0.004 | 0.009 | 0.6869 |  |  |  | -0.002 | 0.01 | 0.7939 | 0.01 | 0.01 | 0.3653 |  |  |
| No | Ref |  |  |  |  |  | Ref |  |  | Ref |  |  |  |  |
| **Birthing Parent Race** |  |  |  |  |  |  |  |  |  |  |  |  |  |  |
| Non-Hispanic Black |  |  |  | -0.56 | 0.01 | **<0.0001** | -0.44 | 0.01 | **<0.0001** | -0.42 | 0.01 | **<0.0001** |  |  |
| Non-Hispanic White |  |  |  | Ref |  |  | Ref |  |  | Ref |  |  |  |  |
| **Birthing Parent Age** |  |  |  |  |  |  | -0.02 | 0.001 | **<0.0001** | -0.02 | 0.001 | **<0.0001** |  |  |
| **Birthing Parent Education** |  |  |  |  |  |  |  |  |  |  |  |  |  |  |
| < High School |  |  |  |  |  |  | -0.29 | 0.02 | **<0.0001** | -0.29 | 0.02 | **<0.0001** |  |  |
| High School/GED |  |  |  |  |  |  | -0.21 | 0.01 | **<0.0001** | -0.21 | 0.01 | **<0.0001** |  |  |
| Some College |  |  |  |  |  |  | -0.13 | 0.01 | **<0.0001** | -0.13 | 0.01 | **<0.0001** |  |  |
| College or More |  |  |  |  |  |  | Ref |  |  | Ref |  |  |  |  |
| **Birthing Parent Marital Status** |  |  |  |  |  |  |  |  |  |  |  |  |  |  |
| Never Married |  |  |  |  |  |  | -0.15 | 0.01 | **<0.0001** | -0.15 | 0.01 | **<0.0001** |  |  |
| Married |  |  |  |  |  |  | Ref |  |  | Ref |  |  |  |  |
| Divorced/Widowed |  |  |  |  |  |  | -0.25 | 0.02 | **<0.0001** | -0.25 | 0.02 | **<0.0001** |  |  |
| **Source of Payment for Delivery** |  |  |  |  |  |  |  |  |  |  |  |  |  |  |
| Private Insurance |  |  |  |  |  |  | Ref |  |  | Ref |  |  |  |  |
| Medicaid |  |  |  |  |  |  | -0.10 | 0.01 | **<0.0001** | -0.10 | 0.01 | **<0.0001** |  |  |
| Self-Pay |  |  |  |  |  |  | 0.25 | 0.03 | **<0.0001** | 0.25 | 0.03 | **<0.0001** |  |  |
| Other |  |  |  |  |  |  | 0.002 | 0.04 | 0.9539 | -0.004 | 0.04 | 0.9083 |  |  |
| **Receipt of WIC During Pregnancy** |  |  |  |  |  |  |  |  |  |  |  |  |  |  |
| Yes |  |  |  |  |  |  | 0.11 | 0.01 | **<0.0001** | 0.11 | 0.01 | **<0.0001** |  |  |
| No |  |  |  |  |  |  | Ref |  |  | Ref |  |  |  |  |
| **Pre-pregnancy BMI** |  |  |  |  |  |  | -0.002 | 0.001 | **0.0020** | -0.002 | 0.001 | **0.0020** |  |  |
| **Parity (including birth on record)** |  |  |  |  |  |  | -0.05 | 0.003 | **<0.0001** | -0.05 | 0.003 | **<0.0001** |  |  |
| **Infant's Sex** |  |  |  |  |  |  |  |  |  |  |  |  |  |  |
| Female |  |  |  |  |  |  | Ref |  |  | Ref |  |  |  |  |
| Male |  |  |  |  |  |  | -0.09 | 0.01 | **<0.0001** | -0.09 | 0.01 | **<0.0001** |  |  |
| **Residential Geographic Region** |  |  |  |  |  |  |  |  |  |  |  |  |  |  |
| Upper Peninsula Prosperity Alliance |  |  |  |  |  |  | 0.12 | 0.03 | **<0.0001** | 0.12 | 0.03 | **<0.0001** |  |  |
| Northwest Prosperity Region |  |  |  |  |  |  | 0.22 | 0.03 | **<0.0001** | 0.22 | 0.03 | **<0.0001** |  |  |
| Northeast Prosperity Region |  |  |  |  |  |  | 0.14 | 0.03 | **<0.0001** | 0.14 | 0.03 | **<0.0001** |  |  |
| West Michigan Prosperity Alliance |  |  |  |  |  |  | 0.05 | 0.01 | **<0.0001** | 0.05 | 0.01 | **<0.0001** |  |  |
| East Central Michigan Prosperity Region |  |  |  |  |  |  | 0.10 | 0.02 | **<0.0001** | 0.10 | 0.02 | **<0.0001** |  |  |
| East Michigan Prosperity Region |  |  |  |  |  |  | -0.05 | 0.02 | **0.0011** | -0.05 | 0.02 | **0.0011** |  |  |
| South Central Prosperity Region |  |  |  |  |  |  | 0.10 | 0.02 | **<0.0001** | 0.10 | 0.02 | **<0.0001** |  |  |
| Southwest Prosperity Region |  |  |  |  |  |  | 0.16 | 0.02 | **<0.0001** | 0.16 | 0.02 | **<0.0001** |  |  |
| Southeast Michigan Prosperity Region |  |  |  |  |  |  | 0.09 | 0.01 | **<0.0001** | 0.09 | 0.01 | **<0.0001** |  |  |
| Detroit Metro Prosperity Region |  |  |  |  |  |  | Ref |  |  | Ref |  |  |  |  |
| **Interaction** |  |  |  |  |  |  |  |  |  |  |  |  |  |  |
| Race*Exposure |  |  |  |  |  |  |  |  |  | -0.06 | 0.02 | **0.0109** |  |  |
| ^a^ Exposed infants were born between 9/1/2015 and 8/31/2016; unexposed infants were born in the same 37-week period in 2013, 2014, or 2015 (9/1/2013-8/31/2014 & 9/1/2014-8/31/2015).  Bolded *P-values* denote statistical significance at α = 0.05.  Sample Sizes Non-Hispanic Black: 32,643 (unexposed), 15,687 (exposed); Non-Hispanic White: 128,639 (unexposed), 62,068 (exposed). | | | | | | | | | | | | | | |

**Table S17. Linear Regression Coefficients for Regression of Size for Gestational Age (Z-Score) on Exposure to the Flint Water Crisis Emergency Declaration in Michigan: Alternate Exposure period (9/1/2015-8/30/2016=exposed) (n=239,037).**

|  | **Model 1** | | | **Model 2** | | | **Model 3** | | | **Model 4** | | | |
| --- | --- | --- | --- | --- | --- | --- | --- | --- | --- | --- | --- | --- | --- |
| **Variable** | **Beta** | **SE** | ***P-value*** | **Beta** | **SE** | ***P-value*** | **Beta** | **SE** | ***P-value*** | **Beta** | **SE** | ***P-value*** |  |
| **Intercept** | 0.05 | 0.003 | **<0.0001** | 0.13 | 0.002 | **<0.0001** | -0.37 | 0.02 | **<0.0001** | -0.37 | 0.02 | **<0.0001** |  |
| **Exposed^a^** |  |  |  |  |  |  |  |  |  |  |  |  |  |
| Yes | -0.02 | 0.004 | <0.0001 |  |  |  | -0.03 | 0.004 | <0.0001 | -0.02 | 0.005 | **<0.0001** |  |
| No | Ref |  |  |  |  |  | Ref |  |  | Ref |  |  |  |
| **Birthing Parent Race** |  |  |  |  |  |  |  |  |  |  |  |  |  |
| Non-Hispanic Black |  |  |  | -0.42 | 0.005 | **<0.0001** | -0.35 | 0.01 | **<0.0001** | -0.34 | 0.01 | **<0.0001** |  |
| Non-Hispanic White |  |  |  | Ref |  |  | Ref |  |  | Ref |  |  |  |
| **Birthing Parent Age** |  |  |  |  |  |  | -0.002 | 0.001 | **<0.0001** | -0.002 | 0.001 | **<0.0001** |  |
| **Birthing Parent Education** |  |  |  |  |  |  |  |  |  |  |  |  |  |
| < High School |  |  |  |  |  |  | -0.25 | 0.01 | **<0.0001** | -0.25 | 0.01 | **<0.0001** |  |
| High School/GED |  |  |  |  |  |  | -0.14 | 0.01 | **<0.0001** | -0.14 | 0.01 | **<0.0001** |  |
| Some College |  |  |  |  |  |  | -0.06 | 0.01 | **<0.0001** | -0.06 | 0.01 | **<0.0001** |  |
| College or More |  |  |  |  |  |  | Ref |  |  | Ref |  |  |  |
| **Birthing Parent Marital Status** |  |  |  |  |  |  |  |  |  |  |  |  |  |
| Never Married |  |  |  |  |  |  | -0.08 | 0.01 | **<0.0001** | -0.08 | 0.01 | **<0.0001** |  |
| Married |  |  |  |  |  |  | Ref |  |  | Ref |  |  |  |
| Divorced/Widowed |  |  |  |  |  |  | -0.13 | 0.01 | **<0.0001** | -0.13 | 0.01 | **<0.0001** |  |
| **Source of Payment for Delivery** |  |  |  |  |  |  |  |  |  |  |  |  |  |
| Private Insurance |  |  |  |  |  |  | Ref |  |  | Ref |  |  |  |
| Medicaid |  |  |  |  |  |  | -0.10 | 0.01 | **<0.0001** | -0.10 | 0.01 | **<0.0001** |  |
| Self-Pay |  |  |  |  |  |  | 0.07 | 0.02 | **<0.0001** | 0.07 | 0.02 | **<0.0001** |  |
| Other |  |  |  |  |  |  | -0.06 | 0.02 | **0.0026** | -0.06 | 0.02 | **0.0023** |  |
| **Receipt of WIC During Pregnancy** |  |  |  |  |  |  |  |  |  |  |  |  |  |
| Yes |  |  |  |  |  |  | -0.06 | 0.01 | **<0.0001** | -0.06 | 0.01 | <0.0001 |  |
| No |  |  |  |  |  |  | Ref |  |  | Ref |  |  |  |
| **Pre-pregnancy BMI** |  |  |  |  |  |  | 0.02 | 0.0003 | **<0.0001** | 0.02 | 0.0003 | **<0.0001** |  |
| **Parity (including birth on record)** |  |  |  |  |  |  | 0.05 | 0.001 | **<0.0001** | 0.05 | 0.001 | **<0.0001** |  |
| **Infant's Sex** |  |  |  |  |  |  |  |  |  |  |  |  |  |
| Female |  |  |  |  |  |  | Ref |  |  | Ref |  |  |  |
| Male |  |  |  |  |  |  | 0.02 | 0.004 | **<0.0001** | 0.02 | 0.004 | **<0.0001** |  |
| **Residential Geographic Region** |  |  |  |  |  |  |  |  |  |  |  |  |  |
| Upper Peninsula Prosperity Alliance |  |  |  |  |  |  | 0.06 | 0.01 | **<0.0001** | 0.06 | 0.01 | **<0.0001** |  |
| Northwest Prosperity Region |  |  |  |  |  |  | 0.07 | 0.01 | **<0.0001** | 0.07 | 0.01 | **<0.0001** |  |
| Northeast Prosperity Region |  |  |  |  |  |  | 0.01 | 0.02 | 0.4491 | 0.01 | 0.02 | 0.4488 |  |
| West Michigan Prosperity Alliance |  |  |  |  |  |  | 0.06 | 0.01 | **<0.0001** | 0.06 | 0.01 | **<0.0001** |  |
| East Central Michigan Prosperity Region |  |  |  |  |  |  | -0.005 | 0.01 | 0.6187 | -0.01 | 0.01 | 0.6195 |  |
| East Michigan Prosperity Region |  |  |  |  |  |  | 0.03 | 0.01 | **0.0006** | 0.03 | 0.01 | **0.0006** |  |
| South Central Prosperity Region |  |  |  |  |  |  | 0.02 | 0.01 | **0.0161** | 0.02 | 0.01 | **0.0162** |  |
| Southwest Prosperity Region |  |  |  |  |  |  | 0.02 | 0.01 | **0.0032** | 0.02 | 0.01 | **0.0032** |  |
| Southeast Michigan Prosperity Region |  |  |  |  |  |  | 0.005 | 0.01 | 0.5327 | 0.005 | 0.01 | 0.5323 |  |
| Detroit Metro Prosperity Region |  |  |  |  |  |  | Ref |  |  | Ref |  |  |  |
| **Interaction** |  |  |  |  |  |  |  |  |  |  |  |  |  |
| Race*Exposure |  |  |  |  |  |  |  |  |  | -0.01 | 0.01 | 0.4606 |  |

^a^ Exposed infants were born between 9/1/2015 and 8/31/2016; unexposed infants were born in the same 37-week period in 2013, 2014, or 2015 (9/1/2013-8/31/2014 & 9/1/2014-8/31/2015).

Bolded *P-values* denote statistical significance at α = 0.05.

Sample Sizes Non-Hispanic Black: 32,643 (unexposed), 15,687 (exposed); Non-Hispanic White: 128,639 (unexposed), 62,068 (exposed).

**Table S18. Predicted Means^a^ and Mean Difference for Birth Outcomes on Exposure to the Flint Water Crisis Emergency Declaration in Michigan: Alternate Exposure period (9/1/2015-8/30/2016=exposed) (n=239,037).**

|  | **Birthweight (Grams)** | | **Gestational Age (Weeks)** | | **Size for Gestational Age (Z-score)** | |
| --- | --- | --- | --- | --- | --- | --- |
|  | **Non-Hispanic Black** | **Non-Hispanic White** | **Non-Hispanic Black** | **Non-Hispanic White** | **Non-Hispanic Black** | **Non-Hispanic White** |
| **Exposure Status^b^** | **Mean (95% CI)** | **Mean (95% CI)** | **Mean (95% CI)** | **Mean (95% CI)** | **Mean (95% CI)** | **Mean (95% CI)** |
| Exposed | 3077.53 | 3327.9 | 38.34 | 38.87 | -0.28 | 0.02 |
|  | (3022.7, 3122.7) | (3316.9, 3338.8) | (38.16, 38.51) | (38.84, 38.91) | (-0.35, -0.21) | (0.002, 0.04) |
| Unexposed | 3240.9 | 3339.6 | 38.38 | 38.87 | -0.25 | 0.04 |
|  | (3232.1, 3249.6) | (3329.1, 3350.1) | (38.21, 38.55) | (38.83, 38.90) | (-0.32, -0.18) | (0.03, 0.07) |
| Least Square Mean Difference | -13.7 | -11.7 | -0.04 | 0.007 | -0.03 | -0.03 |
| (95% CI) | (-18.4, -9.0) | (-16.9, -6.6) | (-0.09, 0.01) | (-0.01, 0.02) | (-0.05, -0.01) | (-0.03, -0.02) |
| *P*-value | **<0.0001** | **<0.0001** | 0.0874 | 0.4241 | **0.0016** | **<0.0001** |

^a^ Linear model (4) stratified by race and exposure status adjusted for covariates: (birthing parent age, education, marital status, source of payment for delivery, receipt of WIC during pregnancy, pre-pregnancy BMI, parity, infant sex, and residential geographic region).

^b^ Exposed infants were born between 9/1/2015 and 8/31/2016; unexposed infants were born in the same 37-week period in 2013, 2014, or 2015.
